# A randomized, controlled pilot study of the effects of vitamin D supplementation on balance in Parkinson's disease: Does age matter?

**DOI:** 10.1371/journal.pone.0203637

**Published:** 2018-09-26

**Authors:** Amie L. Hiller, Charles F Murchison, Brenna M Lobb, Susan O’Connor, Morgan O’Connor, Joseph F Quinn

**Affiliations:** 1 VA Portland Healthcare System, Portland, OR, United States of America; 2 Oregon Health and Sciences University, Portland, OR, United States of America; 3 UC Davis, Sacramento, CA, United States of America; National Health Research Institutes, TAIWAN

## Abstract

**Objectives:**

To explore if short term, high dose vitamin D supplementation is safe and improves balance in persons with Parkinson's disease (PD).

**Methods:**

A pilot randomized, double-blind intervention trial to measure the effects of 16 weeks of high dose vitamin D (10,000 IU/day) on balance as well as other motor and non-motor features of PD. We measured balance, gait, strength, falls, cognition, mood, PD severity, and quality of life before and after 16 weeks of high dose vitamin D supplementation or placebo. All participants also received 1000 mg calcium once daily.

**Results:**

Fifty-one randomized participants completed sixteen weeks of high dose vitamin D supplementation or placebo. The intervention resulted in a rise in serum concentrations of vitamin D (25-OH) (30.2 ng/ml to 61.1 ng/ml) and was well tolerated with no serious adverse events. Serum vitamin D (25-OH) levels rose steadily and did not suggest a leveling off at the end of the 16 weeks. There was not an improvement in the primary endpoint, balance as measured by the Sensory Organization Test (p = 0.43). A post hoc analysis examining treatment effects in younger (ages 52–66) versus older (ages 67–86) participants found a significant improvement in the SOT of 10.6 points in the younger half of the cohort (p = 0.012).

**Conclusions:**

Short term, high dose vitamin D supplementation appears safe in persons with PD, but did not significantly improve balance as measured with the Sensory Organization Test in this pilot study population. A post hoc analysis suggests that vitamin D may have potential for improving balance in a younger population with PD. High dose vitamin D supplementation in PD needs further study especially in light of new research suggesting that mega doses and even moderate doses (as low as 4000IU a day) may increase falls in an older populations.

**Trial registration:**

ClinicalTrials.gov: NCT01119131.

## Introduction

Falls are a major cause of morbidity and mortality in PD. Postural instability, the major cause of falls, is one of the four cardinal features of PD. As PD advances, falls become a major problem and although medications are available for improving the other cardinal features (tremor, rigidity, and slowness of movements), no pharmaceutical or surgical therapy definitively improves balance function. Despite optimal medical therapy, 70% of persons with PD experience at least one fall a year [1–3]. Falls are financially costly with one in four falls necessitating use of health care resources [4].

The cost of falls however is not just financial. Physical mobility is clearly meaningful to persons with PD and is the single most important factor contributing to decline in quality of life [5]. Fear of falling has its own effect and is associated with increased anxiety and depression. This fear can have a major impact, leading to self-induced activity restriction with resultant social isolation and deconditioning [6,7].

Vitamin D deficiency is also prevalent in PD. A cohort study following osteoporosis in men found that 21% of the men with PD were deficient and another 50% were insufficient in vitamin D [8]. In the Atlanta area 55% of persons with PD had insufficient vitamin D levels as compared to 41% of persons with Alzheimer’s disease [9]. Vitamin D deficiency seems particularly prevalent in more advanced PD, with rates as high as 78% in late stages [10]. This is precisely when balance problems are most prominent.

Early studies designed to look at the effects of vitamin D on fractures in non-PD populations found fall rates were lower in those on vitamin D as compared to placebo [11–13]. Four of 5 meta-analyses on this topic show benefits of vitamin D, with odds ratios or relative risks for falls ranging from 0.66 to 0.88 [14–18]. A 2009 meta-analysis showed that higher doses of vitamin D (700–1000 IU per day) were more effective than lower doses (200–600 IU per day) [19]. While the vast majority of studies did not use falls as a primary endpoint, a 2009 study by Pfeifer et al found a 27% decrease in first falls over 12 months in the group receiving 800 IU of vitamin D as compared to placebo [20]. This same group showed improvement in a measure of balance, body sway, with only 8 weeks of vitamin D supplementation [21]. The above was the primary data used in the design of our study. As with many researchers we felt higher doses might offer greater benefit and side effects seemed minimal [22]. However more recent research suggests that in older populations mega doses of vitamin D (300,000 and 500,000 IU given yearly) [23,24] and even doses as low as 60,000 IU a month and 4,000 IU a day [25,26] may results in increased falls and fractures. Most of these studies focused on older populations with mean ages typically being in the mid to late seventies.

We chose to focus on balance rather than leg strength because studies in non-PD population have shown vitamin D-dependent improvement in measures of sway, which is not very strength-dependent [21,27]. Specifically Bischoff-Ferrari saw a 60% decrease in fall rate with vitamin D, and attributed up to 22% of the treatment effect to changes in postural balance and up to another 14% to a changes in dynamic balance. Another study published in 2006 found no improvement in strength after 9 months of vitamin D supplementation, but did find improvements in gait speed and sway [28]. The presence of vitamin D receptors throughout the brain also suggests that there is a central nervous system component mediating the effect of vitamin D upon falls [29].

The Sensory Organization Test (SOT) was selected as the primary outcome measure for this trial based on a cross-sectional pilot study evaluating the relationship between vitamin D levels and five balance related measures (SOT, backwards translation, unilateral stance, sit to stand, and a walk and turn on a narrow platform) [30]. The SOT was identified as the most suited for an intervention study because of: 1) absence of floor or ceiling effect in PD, 2) a relationship in the pilot between scores on the SOT and performance on the pull test commonly used in clinic assessment of balance in persons with PD, and 3) correlation with falls in non-PD populations [31].

The objectives of this pilot study were to explore if high dose vitamin D supplementation is safe in persons with PD and if supplementation improves balance. Post hoc analyses were conducted in order to determine how best to design future studies examining the effects of vitamin D supplementation on balance in PD.

## Methods

### Design

The study was a randomized, double-blind intervention trial to measure the effects of high dose (approximately 10,000 IU/day) vitamin D versus placebo on balance and other motor and non-motor symptoms in persons with PD. All procedures were approved by the Portland VA Medical Center (IRB #2393) and Oregon Health and Science University (OHSU) institutional review board (IRB#6482). Balance, strength, gait, falls, cognition, and mood were measured before and after 16 weeks of high dose vitamin D supplementation or placebo.

### Participants

#### Inclusion criteria

(1) Medically confirmed diagnosis of PD by a movement disorders specialist using the National Institute of Neurological disorders and Stroke (NINDS) criteria. (2) Ability to ambulate 50 feet without the assistance of another person. (3) Ability to cooperate with balance testing. (4) 50 + years of age. (5) Vitamin D (25-OH) level between 21 and 39 ng/ml. (6) Balance dysfunction indicated by: a score of ≥1 on the pull test or 1 fall in last month or 2 near falls in the last month.

We confined the study to persons with low levels of vitamin D (25-OH) based on the hypothesis that persons with lower levels will respond more robustly to supplementation, as shown in other

Studies [32,33]. Subjects with vitamin D (25-OH) levels between 21 and 39 ng/ml were randomized 1:1 to active treatment vs placebo. In order to ensure an ethical standard of care for study volunteers, persons with deficient vitamin D (25-OH) levels (<20ng/ml) were automatically enrolled in the active arm of the study, but both the raters and participants were still blinded to lab results and the intervention.

#### Exclusion criteria

(1) Significant cognitive deficits as defined by a Mini Mental Status Exam (MMSE) of <25. (2) Another neurological or orthopedic deficit that in the investigator’s opinion would have a significant impact on gait and cognition (e.g. stroke, fracture). (3) History of renal stones or renal disease (history of renal transplant, currently on dialysis, or a creatinine > 1.5 at baseline testing). (4) Vitamin D supplementation of > 600 IU a day. (5) Hypercalcemia (based on ionized calcium level). (6) Known untreated tuberculosis infection. (7) Pregnancy. (8) Soy Allergy. All data collection took place and OHSU and the Portland VA Health Care System.

#### Randomization

We used a permuted block randomization scheme to allocate treatments using strata determined by two factors: baseline vitamin D (25-OH) levels (≥20-<30 vs. ≥30-≤40ng/ml) and baseline composite SOT4-6 (<64 vs. ≥64). This is based on what is considered insufficiency of vitamin D and the mean normative SOT score for persons 70–79 years old respectively.[34, 35] Those with < 20 ng/ml baseline vitamin D (25-OH) levels were enrolled into the vitamin D supplementation arm and received 10,000 IU of vitamin D for 16 weeks but both participants and raters were blinded to treatment status. The VA pharmacy, not involved in any other aspects of the study, did the randomization by number using randomization.com. The study coordinator was responsible for participant enrollment.

#### Intervention

Subjects were assigned to one of two groups: (1) placebo plus 1000 mg calcium carbonate or (2) high dose vitamin D plus calcium. In order to approximate 10,000 IU per day with a study drug that contained 13,600 IU per dose, all participants were instructed to take the capsules and calcium carbonate Monday thru Friday. For those in the active arm the weekly dose of vitamin D was 68,000 IU. The dosage given was the same for all participants in the active arm of the study.

The vitamin D was obtained from Capsugel and the calcium carbonate directly from the VA pharmacy (BTR Group Inc is source). The vitamin D placebo was also manufactured by Capsugel and had an identical appearance to the active capsule.

### Outcome measures

All measures were recorded at baseline and after 16 weeks of supplementation except where stated.

Balance Measures: The primary outcome measure was the composite score of static and dynamic balance, as measured by the Sensory Organization Test (SOT) using dynamic posturography. The SOT is carried out using computerized posturography, a system with a moveable surround and platform that contain multiple force plates recording weight transfer. Subjects are placed in a harness and stand on the platform. There are 6 different scenarios. For condition 1–3 the platform is stable and the subject is told to stand as still as they can, first with eyes open (1), then closed (2), then eyes open with the surround in motion (3). For condition 4–6 the base moves in response to the subject’s own motion, termed sway reference. Scenario 4 is with eyes open, scenario 5 is with eyes closed, and scenario 6 is with the surround moving. Each scenario has 3 trials. Weight movement in the anterior posterior direction is rated on a score of 0 (a fall) up to 100.

#### Gait measures

The iMOBILITY device was used to examine gait during a timed up and go test (TUG). Specifically the participant sat in a chair and when told to start, rises, walks to a line 3 feet away, turns, and returns to a seated position. The sensors are interfaced with a computer and record multiple metrics including arm swing, asymmetry, number of steps, and turn duration. We specifically used the turn duration in the analysis.

#### Strength measures

We measured strength of leg flexion and extension using a computerized dynamometer, Biodex. We were able to calculate total work and power using this device [36]. We used total work as the primary strength measure for the analysis.

#### Falls

Subjects were given a diary in which to record their falls at the screening visit. They were instructed to write the time of day of the fall, what they were doing when they fell, if they sustained an injury, and classify it as a fall or near fall. Falls were defined to subjects as unintentionally coming to rest on the ground, or another lower level such as a chair, toilet, or bed [37]. A near-fall was defined as a slip (sliding of the support leg), trip (impact of the swinging leg with an external object) or loss of balance where the person starts to fall but is able to stop or prevent the fall to the ground or other lower surface [38]. Subjects were asked in person every 4 weeks when they met with the study coordinator how often they fell and the diary was reviewed. We had very poor compliance with this portion of the study so the available data was limited.

#### Quality of life (QOL) measures

We used the Nottingham health profile (NHP), a general QOL measure, and the PDQ-39, a PD specific scale [39,40]. Both questionnaires generate a score of 0 to 100. The NHP asks 38 yes or no questions in categories of emotional reactions, sleep, energy, pain, physical mobility, and social isolation. The PDQ-39 has 39 items in categories of mobility, activities of daily living, emotional well-being, stigma, social support, cognitions, communication, and bodily discomfort. Both scales have been validated for use in PD [40]. Because fear of falling also results in limitation we will perform the 16 item Activities-specific Balance Confidence (ABC) Scale at visits 1 and 5 [41]. The ABC scale questions percent confidence (0–100%) subjects have for not losing their balance during a variety of activities. Depression was measured using the profile of Mood States (POMS) questionnaire, a 30-item mood questionnaire.

#### Safety labs

Blood was drawn every 4 weeks to serum check vitamin D (25-OH), ionized calcium, phosphate, and creatinine. Limits for concern were vitamin D (25-OH) above 100 ng/ml, ionized calcium above 1.23 mmol/L, phosphate above 5.5 mg/dL, and creatinine 30% higher than baseline.

#### Possible confounders

Our additional testing for possible confounders included a measure of PD severity—The Unified Parkinson's Disease Rating Scale (UPDRS) motor score [42], a dyskinesia assessment—the Modified Abnormal Involuntary Movements Score (mAIMS), and cognitive testing—Montreal Cognitive Assessment (MoCA), Trail Making A & B, Digit Symbol (WAIS-R), Judgment of Line Orientation, Stroop, Letter-Number Sequencing (WAIS-III), semantic fluency “animals” and “vegetables”, Logical Memory I and II (WMS-R), Boston Naming Test, and Digit Span (WAIS-R).

### Statistical analysis

Because of the pilot nature of the study and the small sample size, a completers’ analysis was performed on the randomized patients. The evaluation of the effect of the vitamin D intervention was carried out using ordinary least-squares (OLS) regression directly on the change in outcome for those assessments completed only at baseline and end of study (i.e. balance, strength, cognitive measures), or with linear mixed effects models for those assessments completed multiple times (i.e. serum vitamin D) in order to account for inter-correlation within subjects.

For all models, the principal contrasts of interest were the effects of the vitamin D intervention on the outcomes by contrasting subjects randomized to vitamin D intervention against the placebo controls to assess the overall vitamin D effect and correlating the on-treatment changes in plasma vitamin D with the changes in study outcomes. We focused on the randomized subjects and did not include those with lower vitamin D levels enrolled only in the active intervention.

Initial exploratory data analysis included simple linear modeling of the outcome variables with the vitamin D intervention with final models correcting for age, gender, disease duration and values at baseline. Given the large number of study outcomes and to account for the multiple comparisons of vitamin D intervention, a Holm-Bonferroni stepwise correction was applied to the sets of p-values from the multiple regression models according to test domain after covariate correction and according to test domain including: clinical and computerized balance, computerized gait, strength, clinical quality of life and mood, executive function, memory, attention, and processing speed. Model integrity was evaluated using standard diagnostic procedures for linear regression models including sensitivity assessment on potential leverage points and assessment of the normality and homogeneity of the outcome residuals.

#### Sample size

We based our power calculation on the primary outcome, the composite SOT (cSOT) score from computerized posturography, for Aim 1. Using our preliminary data on 20 subjects the average cSOT (SD), excluding one score of zero, was 58.5 (19.1) [30]. For conducting a power calculation on the cSOT measure we assume that 10% of subjects will score zero at baseline and follow-up. Among the remaining subjects, we assume SD’s of 19.1 at baseline and follow-up, that the high dose group will have an average score that is 8.8 points higher than at baseline (15% change from 58.5) and the placebo group shows no improvement. There are not studies looking at changes in SOT with vitamin D supplementation, but the Pfeifer did look at body sway, finding a 15% greater improvement in body sway in the intervention versus the control group [21]. With 10% zeros this 15% change among the non-zeros translates to a mean difference in change of 7.9 points. The SD of the changes from baseline depends on the SD’s at baseline and follow-up and on the correlation between baseline and follow-up measures. We hence obtained sample size requirements using a range of correlation coefficients.

Using simulation with PASS 2008 software, we determined the sample sizes needed to detect the 7.9 point mean difference indicated above with 80% power at significance level 0.049 (allowing for an interim analysis at significance level 0.01) for correlations between 0.5 and 0.75, Table 1. The simulation method was based on the Mann-Whitney nonparametric test which is more conservative in this setting than a t-test procedure (that is, using a simple t-test for the simulation would result in smaller required sample sizes). With 0.66 for correlation, we would require 63 subjects per group. Allowing for 10% drop-out we will then plan to recruit 70 subjects per group (this included those with low levels of vitamin D).

**Table 1:** Sample size sample sizes per group needed for 80% power at significance level 0.049 to detect a mean difference in posturography composite change of 7.9.

SD at baseline and at follow-up for composite posturography	Correlation between baseline and follow-up composite posturography	SD of difference between follow-up and baseline scores	Sample size per group
19.1	0.50	19.1	91
19.1	0.66	15.8	63
19.1	0.75	13.5	47

#### Interim analysis

Our planned interim analysis was based on when half of the targeted subjects (including those with vitamin D levels <20ng/ml) have completed the follow-up visit. Specifically we planned to conduct an interim analysis of the primary outcome using a significance level of 0.01. This was felt appropriate to ensure additional safety for patients.

## Results

**Fig 1:**
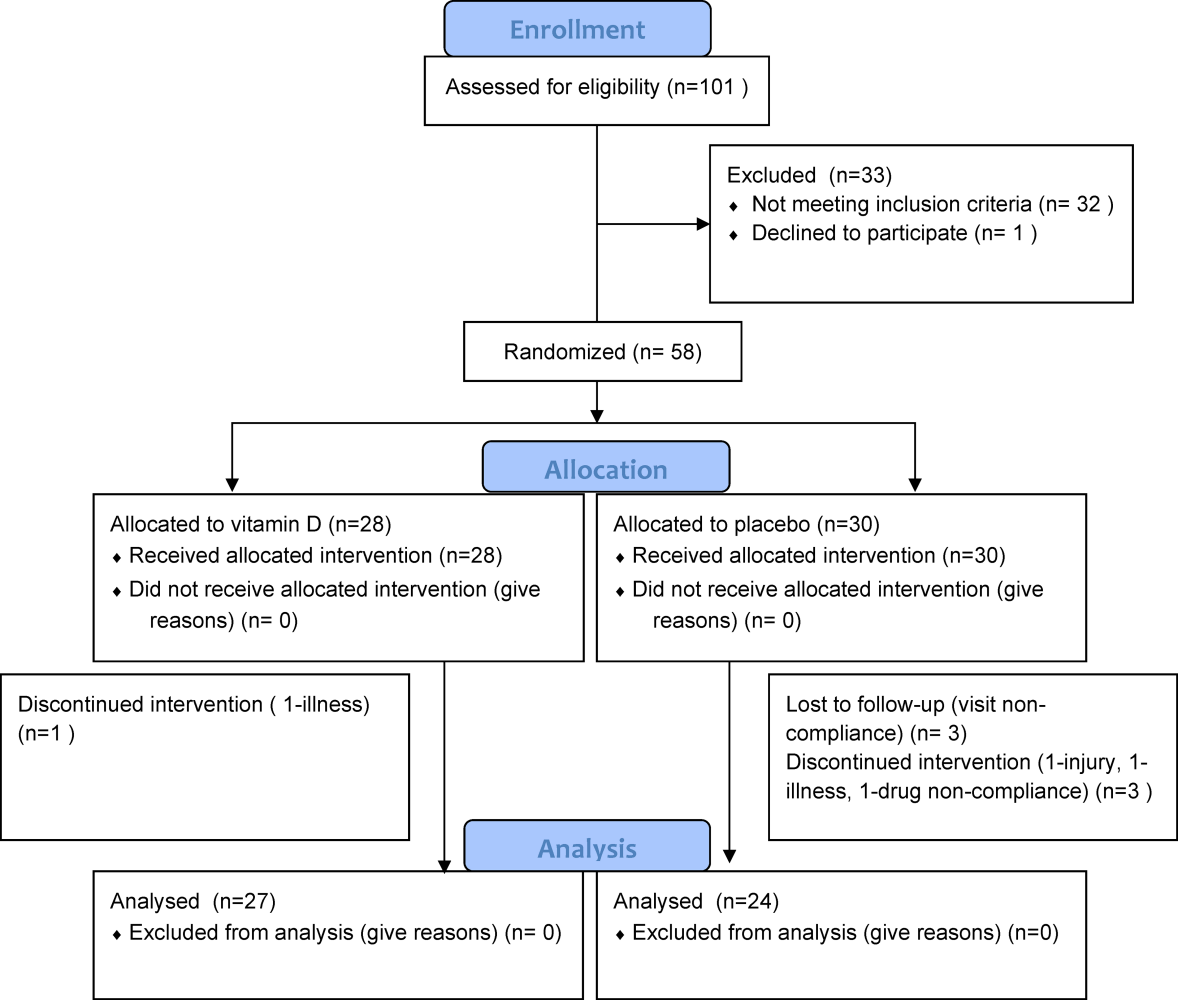
CONSORT diagram. Consort diagram detailing study screening, randomization, and completion.

One hundred and one participants were consented and screened for this study Fig 1. Thirty-three were screen failures (33%), primarily because of vitamin D concentration above 40 ng/ml. There were 68 subjects receiving at least one dose of study drug, mean age (67.2, SD = 8.3), 76.5% male, mean H&Y = 2.3 (SD = 0.4), mean baseline vitamin D = 27.6ng/ml (SD = 7.8), and mean years since diagnosis 9.6 (SD = 6.7). In order to ensure that vitamin D-deficient participants were not denied supplementation, ten participants with baseline vitamin D (25-OH) < 20 ng/ml were not randomized, but were treated with vitamin D and followed according to protocol otherwise. Of the remaining 58 subjects randomized to vitamin D vs placebo, seven were withdrawn by the investigator for visit non-compliance (multiple attempts of phone contact were made prior to withdrawing any subjects) or minor unrelated illnesses. There were 51 randomized participants analyzed in the completers’ analysis. The groups were well matched in terms of age, gender, disease severity, and baseline serum vitamin D levels Table 2. Recruitment started July 2011 and last date of follow-up was January 2015. The trial was stopped after the interim analysis did not show impressive findings and funding was no longer available.

**Table 2:** Baseline demographics.

	Subject Count	All Subjects (N = 51)	Randomized Vitamin D (N = 27)	Placebo group (N = 24)	t-test p-value
Age	51	66.57 (8.07)	64.63 (8.134)	68.75 (7.577)	0.067
Gender (Male)	68	52 (76.5%)	23 (82.1%)	20 (66.7%)	0.262
Hoehn & Yahr	48	2.469 (0.4772)	2.48 (0.4673)	2.457 (0.498)	0.867
UPDRS	51	23.76 (10.61)	22.52 (8.68)	25.17 (12.49)	0.39
Baseline Vitamin D	51	30.14 (5.786)	30.33 (5.378)	29.92 (6.324)	0.802
Levodopa equivalence	27	1025 (595.6)	1194 (694.3)	843.8 (421.3)	0.125
MOCA	50	24.34 (2.967)	23.88 (2.79)	24.83 (3.13)	0.265
Falls Report	39	3.564 (11.54)	4 (15.25)	3.19 (7.407)	0.839

**Fig 2:**
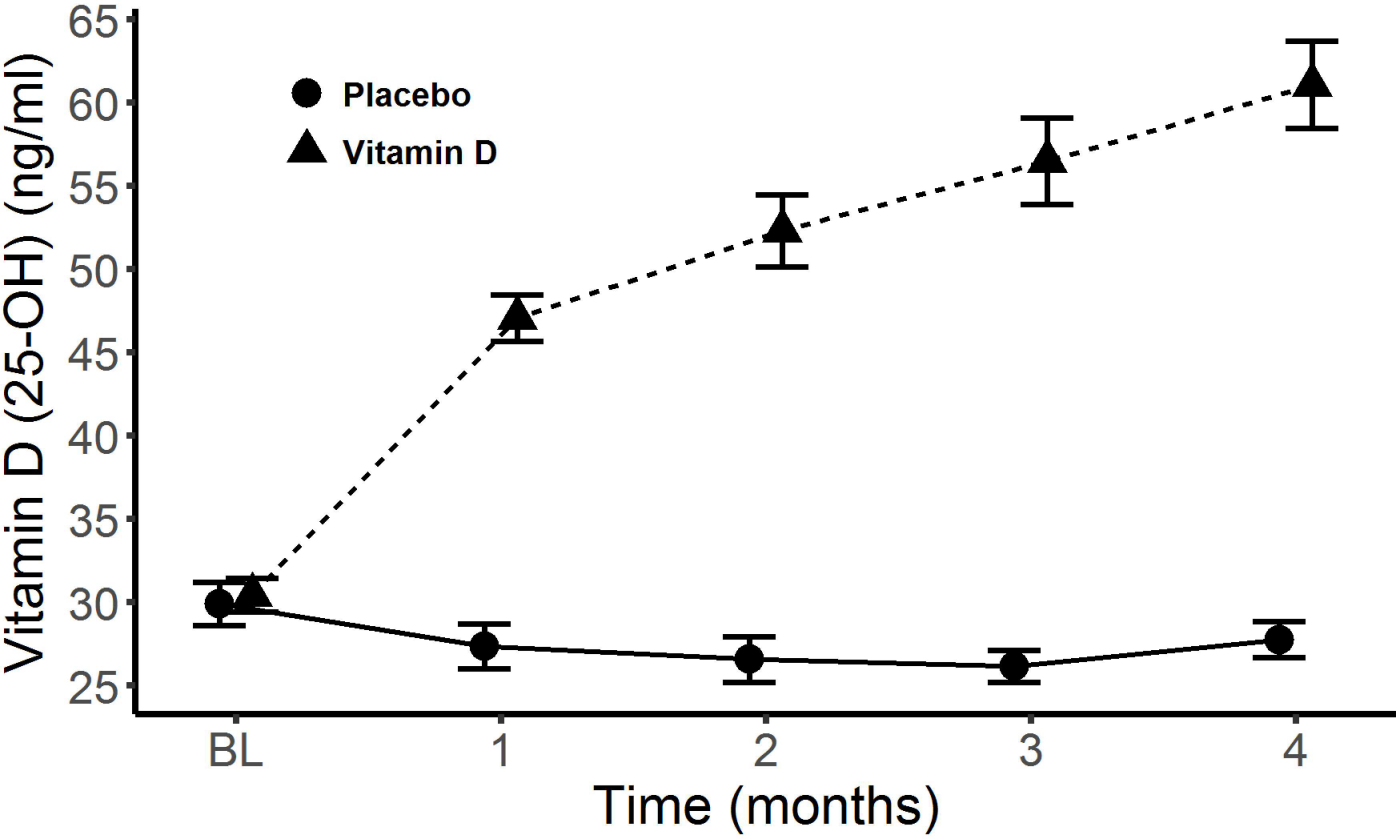
Change in Vitamin D levels. Vitamin D (25-OH) levels in placebo and intervention group over the course of the study.

In the randomized vitamin D group, serum vitamin D (25-OH) concentrations rose from 30.2 ng/ml to 61.1 ng/ml, while in the placebo group serum levels remained fairly stable over the five months of the study, 29.4ng/ml to 27.8 ng/ml Fig 2. In the non-randomized vitamin D group levels rose from 14.7ng/ml to 44.6 ng/ml.

On the primary outcome measure, change in total SOT, there was not a significant improvement in the vitamin-D treated group (p = 0.43) Table 3. There were non-significant trends in favor of vitamin D in verbal fluency (p = 0.157 for vegetables, p = 0.137 for animals), but also a trend in favor of placebo for trails B and trail B-A (p = 0.155 and p = 0.137 respectively). There were no indications of vitamin D effects on secondary measures that examined gait, strength, other aspects of cognition, mood, PD severity, or quality of life Table 3.

**Table 3:** Outcome data for randomized participants.

	**Vitamin D**			**Placebo** **Group**			**t-test** **p-value**
**Gait and Balance Outcomes**	**N**	**Mean (SD)**	**95% CI**	**N**	**Mean (SD)**	**95% CI**	
SOT composite	27	1.741 (11.618)	{-2.641, 6.723}	23	-1.04 (12.91)	{-6.316, 4.236}	0.43
UPDRS-III	27	0.4815 (5.598)	{-1.63, 2.593}	22	-0.1818 (9.733)	{-4.249, 3.885}	0.778
mAIMS Dyskinesia- No Mental Task	27	-0.6296 (2.404)	{-1.536, 0.277}	23	-0.04348 (1.186)	{-0.528, 0.441}	0.271
mAIMS Dyskinesia—Mental Task	25	-1.36 (4.281)	{-3.038, 0.318}	22	0.3182 (2.234)	{-0.615, 1.252}	0.095
BioDex—60 Ext Total Work	26	6.888 (176.4)	{-60.918, 74.694}	22	35.19 (80.74)	{1.451, 68.929}	0.469
BioDex—60 Flex Total Work	26	23.33 (83.16)	{-8.636, 55.296}	22	17.49 (32.02)	{4.11, 30.87}	0.743
iTUG—Median Turn Duration	21	0.09306 (0.5409)	{-0.138, 0.324}	18	-0.0697 (0.6157)	{-0.354, 0.215}	0.39
**Neuropsych Outcomes**	**N**	**Mean (SD)**	**95% CI**	**N**	**Mean (SD)**	**95% CI**	** **
MoCA	26	0.9615 (2.2)	{0.116, 1.807}	24	1.042 (2.51)	{0.038, 2.046}	0.905
Judgment of Line Orientation	26	0.1538 (2.461)	{-0.792, 1.1}	23	0.1739 (2.037)	{-0.659, 1.006}	0.975
PDQ39 Total	24	-0.3333 (10.3)	{-4.454, 3.788}	25	-1.458 (15.66)	{-7.597, 4.681}	0.516
Activities Balance Confidence	24	-0.945 (10.48)	{-5.138, 3.248}	25	2.773 (21.48)	{-5.647, 11.193}	0.862
POMS Total Mood Disturbance	24	-1.083 (11.32)	{-5.612, 3.446}	25	-0.75 (13.66)	{-6.105, 4.605}	0.972
Vegetable Fluency	25	0.44 (3.203)	{-0.816, 1.696}	23	-1.13 (4.224)	{-2.856, 0.596}	0.157
Digit Span—Forwards	25	0.12 (1.922)	{-0.633, 0.873}	23	0.5217 (1.163)	{0.046, 0.997}	0.382
Digit Span—Backwards	25	0.08 (2.019)	{-0.711, 0.871}	23	0.08696 (1.975)	{-0.72, 0.894}	0.99
Boston Naming Task	24	0.5417 (1.382)	{-0.011, 1.095}	23	0.4783 (1.238)	{-0.028, 0.984}	0.869
Animal Fluency	24	0.7917 (3.989)	{-0.804, 2.388}	23	-1.217 (5.018)	{-3.268, 0.834}	0.137
Letter-Number Sequence	24	0.25 (1.984)	{-0.544, 1.044}	23	-0.08696 (2.521)	{-1.117, 0.943}	0.614
Digit Symbol Test	24	-0.6667 (5.947)	{-3.046, 1.713}	21	2 (10.56)	{-2.517, 6.517}	0.314
Trails A (sec)	26	0.4615 (19.65)	{-7.092, 8.015}	23	-0.5652 (20.05)	{-8.759, 7.629}	0.857
Trails B (sec)	23	11.65 (49.03)	{-8.388, 31.688}	21	-17.05 (77.31)	{-50.116, 16.016}	0.155
Trails B-A	23	12.13 (49.83)	{-8.235, 32.495}	21	-17.33 (74.7)	{-49.28, 14.62}	0.137
Immediate Logical Memory Score	21	1 (4.359)	{-0.864, 2.864}	21	1.286 (3.901)	{-0.382, 2.954}	0.824
Delayed Logical Memory Score	22	1.273 (3.731)	{-0.286, 2.832}	21	0.619 (4.522)	{-1.315, 2.553}	0.609

**Fig 3:**
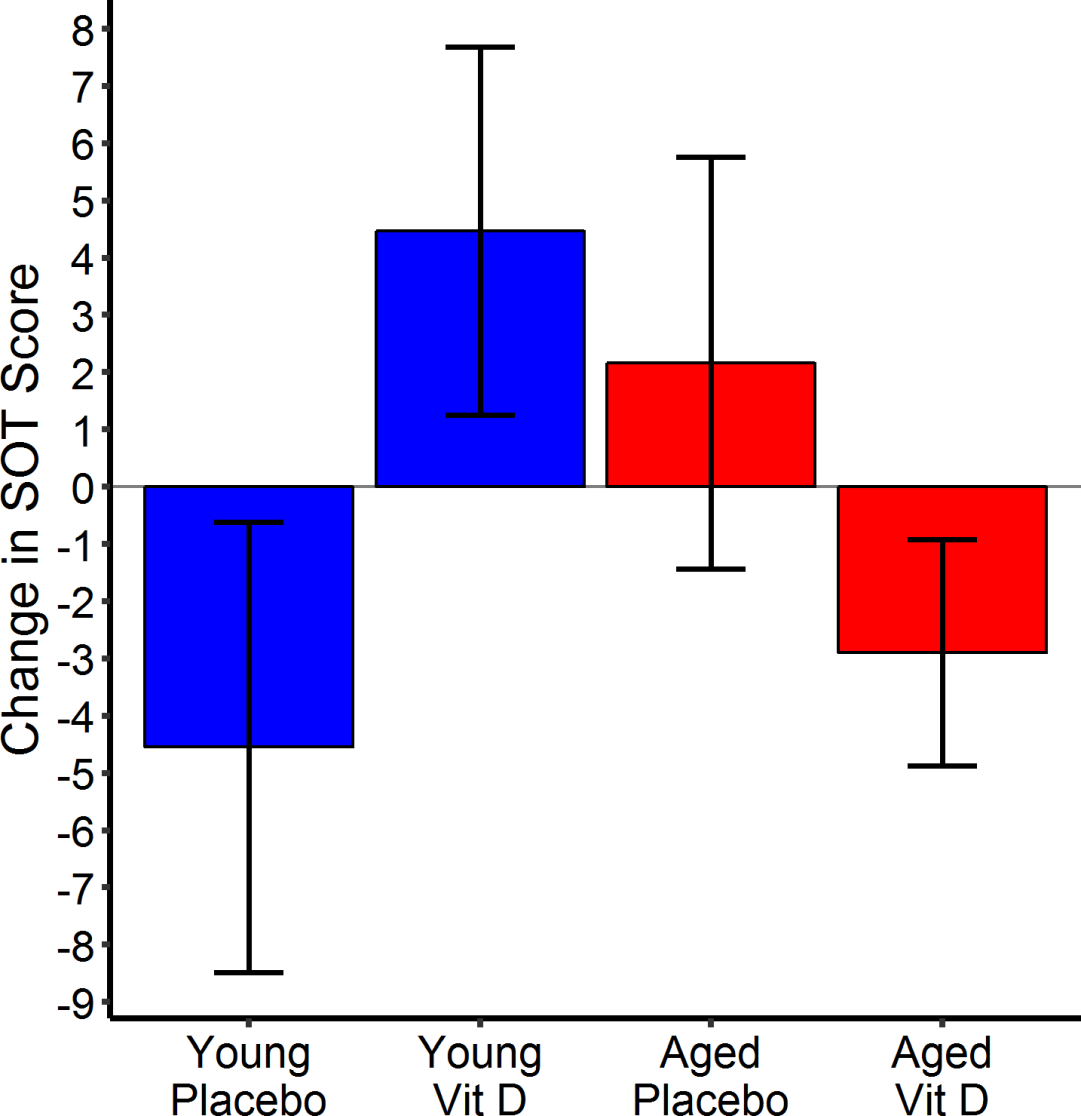
Balance Improvement seen in younger patients. An improvement in balance as measure by SOT was seen in the younger, but not the older, cohort of the study.

However, in a post hoc analysis which divided the groups by median age to those age 67 or older and those 66 or younger, a significant effect of vitamin D treatment was detected on the total SOT score which improved by 10.6 points in the younger vitamin D cohort compared to the older one (t = 2.59, p = 0.012), Fig 3. This post hoc analysis split at the median age did not identify other significant treatment effects of vitamin D. The individuals non-randomly treated with vitamin D were analyzed separately. In this small group (n = 10), no change from baseline in SOT or other relevant outcome measures was appreciated.

The vitamin D intervention appeared safe Table 4. There were no serious adverse events. There were no concerning values in the safety monitoring labs: ionized calcium, phosphate, creatinine.

**Table 4:** Adverse events*.

Adverse Events	Randomized Vitamin D	Placebo	Fisher Test p-value
Any adverse event	12	20	0.32
Serious Adverse Events	0	0	1.0
Hypercalcemia	0	0	1.0
Foot problem (nail fungus, athletes foot, in-grown nail)	3	0	0.97
GI symptoms (indigestion, constipation, food poisoning)	1	3	1.0
Muscle (back sprain, weakness, cramping)	1	2	1.0
Respiratory (Cough/ cold symptoms)	5	6	0.94
Urinary (retention, infection)	0	2	1.0
Procedure (skin biopsy, tumor removal, teeth reconstruction)	0	3	0.97
Other (Lightheadedness, Spider bite, rash, migraine, swollen hand)	0	5	0.92
Infection (elevated WBC source not clear)	0	1	1.0

*There was no difference in total patients with an AE with respect to intervention whether the vitamin D groups were kept separate (p = 0.32) or pooled (p = 0.29) when compared to the placebo group

## Conclusion

This pilot study of vitamin D in Parkinson’s disease found no evidence of a significant treatment effect on any of the primary or secondary outcome measures. The statistical power of the study is admittedly limited but there may nevertheless be some lessons for future studies of vitamin D in PD. For example, the observations that there was no plateau in serum vitamin D in either of the treated groups suggests that 4 months of follow-up may be inadequate for drawing conclusions about either efficacy or toxicity with this type of dosing schedule. The safety profile overall is encouraging, but needs to be considered in light of risks of supra-therapeutic vitamin D levels which have been highlighted by research completed since the initiation of this pilot study. These studies suggest a “U-shaped” response to vitamin D (25-OH), with levels greater than 40–45 ng/ml associated with a paradoxical *increase* in falls and fractures [22]. Animal research suggests that effects of exogenous vitamin D may be different at higher levels, possibly when high enough to suppress parathyroid hormone secretion [43]. It may be important to note that the clinical studies of adverse effects of high dose vitamin D came from trials of individuals over the age of 70 [23–26], which includes only a portion of patients with PD. In this pilot study, the average age was 67, and in fact the post hoc analysis identified evidence of a vitamin D effect upon balance as measured by the total SOT in the younger half of the cohort (mean age 60 years). This finding raises the possibility of an age-dependent role for vitamin D in Parkinson’s disease. It will be important to address the issue of confounding age effects in any future studies of vitamin D in Parkinson’s disease.

## Supporting information

S1 CONSORT checklist (DOC)

S1 Data (XLSX)

S1 Protocol (DOCX)
