# Supplementary material for: A randomized, controlled pilot study of the effects of vitamin D supplementation on balance in Parkinson's disease: Does age matter?
Source: PLoS One. 2018 Sep 26;13(9):e0203637. doi: 10.1371/journal.pone.0203637 (PMC6157857; doi:10.1371/journal.pone.0203637)
Supplement: S1 Protocol — (DOCX) [file pone.0203637.s003.docx]

**Research Plan**

**Objectives:** Based on multiple meta-analyses which show that vitamin D supplementation decreases falls in the elderly we plan to examine the effects of vitamin D supplementation on balance and falls in persons with PD.^8-12^ This project has both training value and great clinical significance for veterans with PD.

**A.1 Objective 1:** To determine if high dose vitamin D supplementation improves balance in persons with PD.

We hypothesize that vitamin D supplementation will improve balance function in persons with PD. Participants will undergo 16 weeks of supplementation with calcium plus high or placebo dose vitamin D. The primary outcome measure will be change in the static and dynamic balance (as measured by computerized posturography) with placebo and high dose vitamin D.^13^

**A.2 Objective 2:** To determine whether vitamin D affects balance primarily through effects on strength or on the central nervous system.

We hypothesize that vitamin D supplementation will at least partially improve balance through effects on the central nervous system. Since an effect of vitamin D upon muscles and strength is established, we will determine the proportion of improvement of balance performance attributable to strength.^14^ We hypothesize that changes in strength will not account for all the improvement seen in balance. By measuring three different areas of balance we will be able to determine if supplementation has effects on static, dynamic, and/or ambulatory balance and the contribution of change in strength to each of these.

**A.3 Objective 3:** To determine if vitamin D supplementation improves balance in a clinically meaningful way.

We hypothesis that vitamin D supplementation will improve balance in a clinically meaningful way. This aim is intended to determine if changes detected by highly sensitive posturography instruments are also clinically important by measuring quality of life with the Nottingham Health Profile (NHP) and the 39-item Parkinson’s Disease Questionnaire (PDQ-39) and balance confidence with the Activities-Specific Balance Confidence (ABC) scale.^15, 16^ Research shows decline in physical mobility is the single most influential factor on measures of quality of life in persons with PD.^17^ In addition fear of falling often leads to self-induced activity restriction, declines in mobility status, and subsequent decline in emotional well being.^18-20^ If vitamin D improves balance in a clinically meaningful way quality of life and balance confidence should also improve.^21^

**B. Background and Significance**

**B.0 Introduction**

Although vitamin D has traditionally been associated almost entirely with bone health, both clinical and basic science findings over the last two decades suggest vitamin D plays a role in the nervous system. The most impressive finding from clinical research consists of a number of studies aimed at elucidating the role of vitamin D in minimizing fractures due to falls.^8-12^ These studies surprisingly showed that therapeutic vitamin D levels not only reduced fractures but also falls in the elderly, suggesting a direct effect of vitamin D on the aging nervous system. The mechanism underlying this effect on falls remains uncertain, but effects on balance via muscles or the central nervous system are plausible based on studies of the distribution of vitamin D receptors.^22^

The primary goal of this study is to determine if vitamin D improves balance in PD. A secondary goal is to better understand the effects of strength versus the central nervous system on balance performance using state of the art technology to assess static, dynamic, and ambulatory balance. The training aspect of this proposal is to develop expertise in sophisticated methods for assessing balance and in conducting clinical trials to improve balance. These skills will permit Dr. Hiller to transition to an independent researcher working further with vitamin D or other candidate interventions to improve balance in PD or other at risk populations.

**B.1 Balance and Parkinson’s disease**

Falls: Falls are a major cause of morbidity and mortality in PD. Postural instability, the major cause of falls, is one of the four cardinal features of PD; the other three being tremor, rigidity, and slowness of movements. As PD advances falls become a major problem and although medications are available for improving the other cardinal features, no medical or surgical therapy definitively improves balance function. Despite optimal medical therapy, 70% of persons with PD experience at least one fall a year.^23-25^ Falls are financially costly with one in four falls necessitating use of health care resources.^26^ The cost of falls however is not just financial.

Effects on Quality of Life: Physical mobility is clearly meaningful to persons with PD and is the single most important factor contributing to decline in quality of life.^17^ Fear of falling has its own affect and is associated with increased anxiety and depression. This fear can have a major impact, leading to self-induced activity restriction with resultant social isolation and deconditioning.^27, 28^

The nature of balance problems in PD: Early on in PD, falls are fairly uncommon and balance and gait appear fairly normal, unless very rigorous testing is done.^29^ Most falls in PD appear to be due to a primary balance disorder rather than an environmental factor, such as tripping over an object on the floor.^24, 30^ Many falls are a consequence of attempts to change posture such as arising from a chair or turning. Freezing of gait, when a person with PD cannot get his or her legs to move, is also a common cause of falls as the disease progresses.^25^ Both balance and freezing of gait are not likely related to changes in the dopaminergic system that seems to be responsible for most of the other motor symptoms in PD. Degeneration in other brain areas, possibly the adrenergic locus coeruleus or the cholinergic/glutaminergic pedunculopontine nucleus cause mobility problems associated with PD.^25^ This explains why interventions targeting the dopaminergic system are ineffective for balance problems.

**B.2 Candidate interventions to improve balance in PD**

Pharmaceuticals: There are no effective medications to improve balance even in PD. The most obvious, dopaminergic medications, do not appear particularly beneficial for balance and falls. A prospective study found that falls in PD more commonly occurred in the “on” dopamanergic state and multiple studies showed little or no improvements on postural reaction when dopamine was given.^24, 31-33^ Small studies of norepinephrine precursor (l-threo-dopa), methylphenidate, and donepezil suggest some benefits but there are no randomized clinical trials.^34-37^

Surgical - deep brain stimulation (DBS): Multiple reviews of DBS side effects list postural instability and gait disorders as complications in over 10% of subjects.^38, 39^ In the VA cooperative study there were significantly more problems with falls and gait disturbances in the DBS as compared to the medical management group. Increased falls were present in those with DBS at 3 and even at 6 months post-surgery.^40^

Physical therapy: Prior to 2008, systematic reviews and meta-analyses on the effects of physiotherapy on falls in PD were inconclusive.^41, 42^ A 2008 meta-analysis focused solely on exercise based programs did find evidence for an improvement in balance and gait speed, but data was insufficient to comment on effects on falls.^43^ Rigorous treadmill training programs and other interventions (i.e. tango and tai chi) appear to have some benefit.^44-47^

In summary, currently available medical, surgical, and rehabilitative treatments for PD are not affective for the treatment of balance impairment, which consequently impacts quality of life and causes untreatable disability late in the disease. An affordable, safe, accessible, non-dopaminergic intervention for improving balance in PD could have substantial effects on quality of life and cost of care for later stage PD. We hypothesize that vitamin D supplementation may represent such an intervention.

Vitamin D: The idea to look at vitamin D for improving balance in PD comes from studies in the late 1990’s and early 2000’s. Studies designed to look at the effects of vitamin D on fractures found fall rates were lower in those on vitamin D as compared to placebo.^48-52^ Four of 5 meta-analyses on this topic show benefits of vitamin D with odds ratios or relative risks for falls ranging from 0.66 to 0.88.^8-12^ At least one study has shown that high doses (700 IU or more per day) are more effective than standard doses.^53^ The vast majority of studies also did not use falls as a primary endpoint. A 2009 study did use falls as the primary outcome, finding a 27% decrease in first falls over 12 months in the group receiving 800IU of vitamin D as compared to placebo.^54^ This same group showed improvement in a measure of balance, body sway, with only 8 weeks of vitamin D supplementation.^55^ The NIH conference “Vitamin D and Health in the 21st Century: an Update” stated “supplemental vitamin D and calcium might reduce the risk of falls” and identified determining the mechanism through which it does so as a key research need.^56, 57^ The goal of the study proposed here is 2 fold; (1) to see if vitamin D supplementation improves balance performance in a specific population at high risk for falls and vitamin D deficiency, persons with PD and (2) to better characterize the contribution of strength versus the central nervous system on balance improvements with vitamin D supplementation.

**B.3 Vitamin D**

Vitamin D and bone health: The medical community knew the importance of vitamin D in bone health almost a century ago. Rickets, the classic disease of soft bones seen in children with vitamin D deficiency, was documented as far back as the second century AD.^58^ Most of what is known about vitamin D is based on bone health, but recent research has demonstrated effects of vitamin D on the muscular, immune, endocrine, and central nervous systems.^14, 57^ Vitamin D in humans is primarily obtained via the skin, with less via the diet primarily from oily fish, fortified foods, and supplements. In the skin 7-dehydrocholesterol is converted to previtamin D3 and then quickly to D3 in a heat dependent process.^59^ In the diet vitamin D is in the form of D2 or D3. Both forms are converted in the liver to 25-hydroxyvitamin D. This inactive form is what is generally measured in the serum. For bone metabolism the kidneys are the primary point of conversion into the active form, 1,25 dihydroxyvitamin. This step is regulated primarily by phosphorus, calcium, parathyroid hormone, and fibroblast growth factor 23 (FGF-23). The active form of vitamin D affects bone health by increasing calcium and phosphorus absorption in the intestines and the conversion of preosteoclasts into mature osteoclasts. Active D also decreases parathyroid hormone (PTH) providing negative feedback (PTH stimulates the kidney to convert more vitamin D into the active form). Recommendations regarding vitamin D levels and supplementation are generally based on bone health; using PTH as an index. Elevated PTH indicated inadequate vitamin D. PTH and vitamin D are inversely associated until vitamin D levels are 30-40ng/ml, at which point PTH levels are normal.^60^ Traditionally vitamin D deficiency is defined as less than 20ng/ml, insufficiency as less than 30ng/ml.

α-Hydroxylase (1 α -OHase), the enzyme that converts vitamin D to its active form, is present in osteoclasts and also in: skin, macrophages, placenta, colon, prostate, endothelium, parathyroid glands, muscle, and brain. Vitamin D receptors (VDR) are present in the traditionally known location related to bone metabolism: enterocytes, osteoblasts, distal renal tubules. But more recently VDRs have been found in parathyroid gland cells, skin keratinocytes, promyelocytes, lymphocytes, colon cells, pituitary gland cells, ovarian cells, muscle, and again brain.^57^

With new locations and functions of vitamin D being discovered the definition of sufficient and insufficient levels are in question. The recommended daily allowance (RDA) of vitamin D for persons 51-70 years old (600 International Units (IU) and for those over 71 (800 IU) is felt by some investigators to be much too low for optimal health outcomes.^61^ Many investigators recommend minimal supplementation of 1,000 IU a day and feel long term supplementation as high as 10,000 IU a day is safe.^62,^ ^63^ The most common toxicity related to supplementation is from resultant hypercalcaemia causing nausea and vomiting, loss of appetite, excessive thirst, frequent urination, constipation, abdominal pain, muscle weakness, muscle and joint aches, confusion, lethargy and fatigue. Vitamin D toxicity is very rare and seems to produce nonspecific symptoms of nausea, vomiting, poor appetite, constipation, weakness, and weight loss.^64^ Data is limited in humans but levels consistently above 200ng/ml are considered potentially toxic.^62^ More in-depth animal studies have not indicated toxicity in levels under 400ng/ml.^65^

Vitamin D and strength: Vitamin D likely affects falls at least partially through improvement in strength. Known for many years, severe vitamin D deficiency can cause a myopathy.^14^ Activation of vitamin D receptors (VDRs) in muscle leads to protein synthesis, muscle cell growth, and improved muscle function.^66^ Improvement in strength of knee extension was seen after 3 months with greater improvement after 6 months of supplementation in a group of vitamin D deficient women.^67^ Another study looking at an indirect measure of muscle strength (time to stand) found decreases in time as D levels increased with a maximum effect at approximately 48ng/mL serum D levels.^68^ The role that changes in strength play balance and falls reduction however is a less clear.

Balance and strength: There is a great deal of epidemiologic data that indicates that weakness in a risk factor for falling; however few studies examine the relationship between balance and strength.^69, 70^ A study in persons with in polio showed a strong association between strength and balance on a foam surface, but not a very good association on a solid surface.^71^ Studies in elderly fallers also indicated more of an association between strength and balance with more difficult balance testing, i.e. eyes closed on a solid surface or eyes opened on foam.^72, 73^ Carter et. al. demonstrated that 10% of variance in static balance and 26% in dynamic balance was accounted for by strength.^74^ Progressive resistance training in isolation has not been shown to improve balance.^75^ In regards to falls the role of strength again is unclear. A review of 36 intervention studies found ones including strength training as a component of a multifactorial intervention overall had the best influence on falls reduction, but this was unrelated to an improvement in strength.^70^

Vitamin D and balance: We hypothesis that vitamin D also affects balance through the central nervous system. We expect improvements in balance that are not accounted for by changes in strength. The presence of vitamin D receptors throughout the brain suggests the likelihood that there is a central nervous system component to vitamin D reducing falls.^22^ Two studies focused specifically on balance changes with vitamin D supplementation, showed improvement in measures of sway, which is not very strength dependent.^55, 76^ The Bischoff-Ferrari paper saw a 60% decrease fall rate and attributed up to 22% of the treatment effect to changes in postural balance and up to another 14% to a changes in dynamic balance. Another study published in 2006 found no improvement in strength after 9 months of vitamin D supplementation, but did find improvements in gait speed and sway.^77^

**B.4 Vitamin D and Parkinson’s disease**

Vitamin D deficiency is prevalent in PD. Our data from the MrOS study found that 21% of the men with PD were deficient and another 50% were insufficient in vitamin D.^4^ Vitamin D deficiency is more common in PD than in AD. In the Atlanta area 55% of persons with PD had insufficient vitamin D levels as compared to 41% of persons with AD.^78^ Vitamin D deficiency seems particularly prevalent later in disease, with rates as high as 78%.^79^ This is precisely when balance problems are most prominent. It is not entirely clear if vitamin D metabolism is unique in persons with PD, but deficiency is clearly prevalent.

A central mechanism through which vitamin D could affect balance in elderly fallers is not well understood. The presence of vitamin D receptors and the final converting enzyme in vitamin D metabolism in the brain suggests a role in CNS function. The beneficial effects of vitamin D may be a general effect on neurons through its up-regulation of neurotrophic factors such as GDNF.^80, 81^ In terms of the regions postulated to be responsible for the gait and balance problems in PD, animal studies show protection of the locus coeruleus (with a predominance of noradrenergic neurons) by systemic vitamin D which attenuated iron-induced oxidative injuries in rats.^82^ In cortical cell cultures, vitamin D pretreatment decreased cyanide-induced cell death which seemed to be through up regulation of UCP-2 (uncoupling protein-2) resulting in reduced mitochondrial protein leak and stabilization of mitochondrial function.^83^ Other cell population may be similar, but are not yet been studied or reported in the literature. The Ibi study found protective effect from subsequent insult was only present when vitamin D was given at least 8 hours prior to the toxic insult, suggesting protein synthesis is required.^84^ The particularly high prevalence of vitamin D receptors in the substantia nigra (likely on dopamine cells) the primarily area of pathology in PD is of obvious interest. Eyles et. al. found vitamin D receptors were present in 42 brain areas but only stained at the highest level within the substantia nigra.^22^ Even though the dopaminergic system may not be responsible for balance problems in PD, it is possible vitamin D has affects on motor symptoms such as tremor, rigidity, and slowness.

In summary, evidence for vitamin D receptors, converting enzymes, and binding proteins in many areas of the CNS implies that vitamin D acts directly on the CNS. Although data from this study will not determine the specific brain regions or mechanisms affected by vitamin D, the data generated will determine if an effect beyond the muscle is evident, and may be used to generate more specific hypotheses regarding localization and mechanism to pursued in future studies.

**B.5 Gait and Balance Evaluation**

We will evaluate balance by measuring static standing balance (stance on a static platform), dynamic standing balance (stance on a moving platform), and ambulatory balance (rising from a chair, walking, and turning). Static and dynamic balance will be measured with computerized posturography.^13^ This device records postural sway under both static and dynamic conditions during the sensory organization test (SOT) (see D.5.1). Because we have one of only 8 research level machines in the world we are able to achieve additional raw data and analyze it at a more in-depth level, thereby improving sensitivity. Research by Dr. Horak showed that movement in the medial lateral direction is more sensitive than movement in the anterior posterior direction (used in the SOT score).^85^ We have also found this to be true in our pilot data and will calculate the root mean square of the velocity in the medical to lateral direction (see C.4.2). In a recent study by Horak et al. the root mean square velocity in the medial lateral direction was abnormal even in very early PD, when traditional measures of balance were normal.^86^

Ambulatory balance will be measured via a timed up and go (TUG). This is a widely used test of balance and gait but again because of the technology available at OHSU we will be able to evaluate the TUG in a much more sophisticated manner. We will use the iMOBILITY device, developed through collaboration between OHSU and Portland State University, which records over 50 metrics related to the TUG (see C.4.3).^87^ Turn velocity will be the primary measure used for this study because it appears to be very sensitive in PD.

Both posturography and iMOBILITY have been shown reliable in PD. By comparing the performance on the various tests before and after intervention with vitamin D supplementation we will be better able to characterize any changes in balance. We will also determine any changes in strength using the Biodex system to determine total work performed during leg flexion and extension (see D.5.3).^88^ The pattern of results with the posturography and iMOBILITY may suggest which structures in the central nervous system are affected by vitamin D.

**B.6 Relevance to Veterans**

Balance problems and resultant falls are a major issue for person with PD as well as many other veterans. Every year almost half the population over 65 year of age experiences a fall.^114^ A recent review of the vitamin D levels I persons with PD in VISN 20 indicated that only 7.5% of patients with PD had ever had levels checked and among those less than 20% had sufficient levels.^5^ If higher vitamin D levels improve balance, inexpensive supplementation may be able to decrease fall rates and their associated morbidity and mortality. This has the potential to both improve care and decrease cost of care for these patients.

**B.5 Summary of Background**

Falls and balance problems are common in PD and costly both financially and in terms of quality of life. There are few interventions that improve balance in PD and data on non-PD populations suggests that vitamin D reduces falls. Vitamin D plasma concentrations are low in a substantial portion of PD patients. These observations justify investigating vitamin D as a candidate intervention for balance difficulties in PD. In addition because of the unique resources available in Portland we have the equipment and expertise to look at the effects of vitamin D on balance in a more sensitive manner. By using a regression analysis we will determine the relative contribution of strength to static, dynamic, and ambulatory balance. The training aspect of the grant is immense. It will provide Dr. Hiller experience with state of the art technology and world experts in an area with great research needs.

**C.0 Work Accomplished**

**C.1 Vitamin D levels are low in men with PD:** Dr. Hiller examined vitamin D levels in men with and without PD, using data from a large cohort study, The Osteoporotic Fractures in Men (MrOS) study. Subjects are men aged 65 and older from 6 locations throughout the United States. At baseline vitamin D levels were measured in a randomly selected subset (n=1495) of participants including 14 of 52 men who identified themselves as having physician-diagnosed PD. Of the men with PD, 71% (10) had either insufficient or deficient levels of Vitamin D. Vitamin D levels were similar in men with PD compared to controls; 25.3 (24.8-25.6) ng/ml vs. 25.4 (21.3-29.6) ng/ml. The results suggest vitamin D deficiency is common in community dwelling men with PD. Dr. Hiller presented this poster at a national meeting sponsored by the Parkinson’s Study Group.^4^

**C.2 Vitamin D and osteoporosis are under assessed in Veterans with PD:** Dr. Hiller examined the frequency of vitamin D and bone density testing over a 5 year period (from August 2003 to August 2008) among veterans with PD in the Veterans Integrated Service Network (VISN) 20. Of 3,128 persons carrying a diagnosis of Parkinson's disease only 236 or 7.5% had a vitamin D level checked during the five year period assessed. Even fewer, 129 or 4.1%, had bone density testing. This is in spite of the fact that over 75% of these patients were over the age of 70 (the age routine bone density assessments are recommended for men by the National Osteoporosis Foundation).^89^ The mean vitamin D level was 26.6 ng/ml (95% CI of 24.7-28.4 and 73% were either insufficient or deficient. The data show that osteoporosis and its risk factors in PD patients are vastly under assessed within the VA system. Dr. Hiller presented this poster at the 2009 Movement Disorder’s Society Annual Meeting.^5^


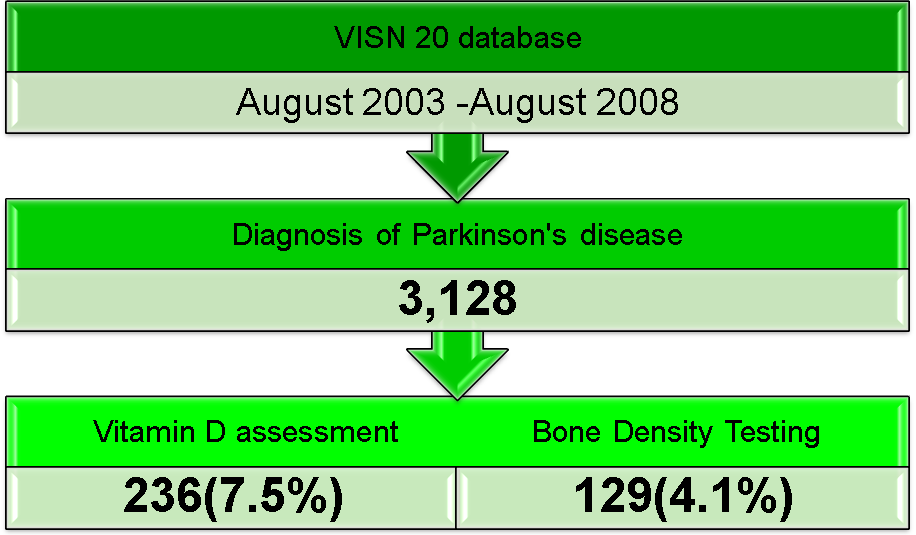


**Vitamin D & Bone Density Testing in**

**VISN 20 Patients with PD**

**Figure 2:** shows how rarely vitamin D and bone density testing are done in men with PD in the VISN 20 system.

**Figure 1:** shows the percentage of men with deficient, insufficient, and sufficient vitamin D levels based on their PD status. Over 70% of men with PD were either deficient or insufficient in vitamin D.

**C.3 Vitamin D levels are correlated with mobility in community dwelling elderly:** Vitamin D levels were correlated with falls, loco-motor function, and cognitive function in subjects from the Intelligent Systems for Assessment of Aging Changes Study (ISAAC). This is a study of adults over age 70 who have computerized equipment in their homes that monitor their movement. They also perform regular computerized questionnaires and come in for periodic testing. Mean vitamin D was 38.0ng/ml (range=9-90 ng/ml). In the 6 months surrounding the vitamin D testing (3 months before and after), 24% (n=37) reported at least one fall. The mean vitamin D of non-fallers was significantly greater 39.7ng/ml (SD 15.3), than both one-time fallers 34.1ng/ml (SD 9.8), and multiple-fallers 28.6ng/ml (SD 11.8) (p=0.03). Vitamin D was also greater in subjects with higher MMSE scores; 42.8±15ng/ml for MMSE=30 (n=42), 36.7±14.4ng/ml for MMSE=27-29 (n=89), and 34.8±13.1ng/ml for MMSE 22-26 (n=21) (p=0.05). Greater plasma vitamin D levels are associated with fewer falls and higher MMSE scores. Dr. Hiller is responsible for the developing the analysis design in collaboration with a research associate and for the drafting of the abstract and future manuscript. This abstract has been submitted for the 2010 American Academy of Neurology Meeting.

**Figure 4:** shows that the SOT composite score (SOTc) correlated better with performance on UPDRS Postural Response score [normal (0), mildly affected (1) and severely affected (2)] than backwards translation (BT) or sit to stand (S2S).

**Figure 3:** shows the average serum vitamin D levels for non-fallers, single fallers, and multi-fallers. Non-fallers had the highest levels and multi-fallers the lowest.

**C.4 Computerized posturography is sensitive to balance deficits in PD**

C.4.1 Candidate balance tests: Balance testing data itself is important in the design of this study. One of the primary aims of the pilot balance study was to select appropriate balance tests for the intervention study. Dr. Hiller has enrolled and tested 35 of the 40 subjects planned for the pilot study using 5 posturography tests. The five tests were:

(1)Sensory Organization Test (SOT): The SOT measures sway during 6 scenarios. In 1-3 the base is stable and eyes are open, then closed, and then the visual surround moves. In 4-6 the base moves and the subject has eyes open, then closed, then the visual surround moves. It generates a score of 0 (fall) up to 100 for each scenario and an overall composite score (see D.5.1).

(2)Backwards Translations (BT): Measures latency, symmetry, amplitude scaling, and force of reactions to posterior displacement. The platform upon which the subject stands quickly moves backwards to elicit the reactive response.

(3)Unilateral Stance with Eyes Open and Closed: This test quantifies mean sway velocity and symmetry of standing on one leg with eyes open and closed.

(4)Sit to Stand (S2S): Measures the time to move the center of gravity (COG) over the feet, the amount of force exerted by the legs during the rise phase, the sway velocity (COG sway) and the weight symmetry during the sit to stand task and for 5 seconds after arising.

(5)Walk and Turn: Measures the amount of time to complete a 180 degree turn to the right and then to the left on a narrow platform. It also measures COG during right and left turns.

The balance data on the initial 20 subjects has been analyzed. Several of the tasks were too difficult for the majority of subjects to perform safely (unilateral stance and walk and turn). Subjects consistently performed well on backwards translations and sit to stand; improvements would not be seen with any type of intervention because of a ceiling affect. The SOT stood out as the best test that would be sensitive to changes in balance. Figure 4 shows the relationship between the Unified Parkinson’s Disease Scale (UPDRS) postural response score (also called a pull test) and the 3 posturography tests that the majority of subjects were able to perform (SOT, backwards translation, and sit to stand).

C.4.2 SOT is sensitive to balance impairments in PD: The SOT stood out by far as the most sensitive test of balance impairment in PD. Scores for the SOT range from 0 for a fall to a ceiling of 100, representing perfect stability. Of the various tests used in the pilot study, the SOT is the most widely reported in the literature and has been shown to correlate with falls.^90, 91^ Figure 5 categorizes subjects based on postural response score of the motor UPDRS, showing subjects SOT scores for scenarios 1-6 and the overall composite score. The average

**Figure 5:** shows the 6 SOT scores for PD patients whose UPDRS Postural Response score was normal (0), mildly affected (1) and severely affected (2) during the 6 conditions and composite. The best possible score is 100; a fall is 0.

**Figure 6:** shows the root mean square of velocity in the medial-lateral direction for the 6 SOT scenarios for PD patients whose UPDRS Postural Response score was normal (0), mildly affected (1) and severely affected (2). A lower score corresponds to better balance, falls were rated as 50.

score is higher in the group with normal response on the pull test, with progressively lower scores on SOT as the pull test performance worsens. Another measure that has been shown more sensitive to balance problems in PD, the root mean square of the velocity in the medial lateral direction shows a similar relationship (see Figure 6).^86^

C.4.3 iMOBILITY is sensitive to ambulatory balance deficits in PD: iMOBILITY has not been used in the pilot study. In performing the pilot study Dr. Hiller realized that one key aspect of balance that was not measured with the battery in the pilot study was ambulatory balance. This is particularly important in PD because of freezing which is only evident when a person ambulates. The walk and turn had the potential to demonstrate ambulatory imbalance but because of the shape and size of the posturography platform used, most of the subjects could not complete the test safely. By adding iMOBILITY, ambulatory balance will be included in the analysis. Although Dr. Hiller does not have personal experience with this test, Dr. Horak and her team have used the device in persons with PD. The specific measure used will be turn duration as measured during a timed up and go using iMOBILITY. Figure 7 from Dr. Horak’s data shows the increase in turn duration time as disease progresses in persons with untreated PD.

**Figure 7:** shows that turn duration increases as PD progresses. Turning takes longer for persons with PD as compared to controls.

**Turn Duration as Measured by iMOBILITY in Parkinson’s Patients and Controls**

**D.0 Work Proposed: Research Design and Methods**

**D.1 Overview of Design:** This proposed study is a randomized, double-blind intervention trial to measure the effects of high dose (10,000 IU/day for 5 days a week) vitamin D versus placebo on balance and falls in persons with PD. We will recruit 140 subjects with idiopathic PD and measure static, dynamic, and ambulatory balance, strength, and falls before and after high or placebo dose vitamin D supplementation. The following will be obtained at the screening visit: balance measures, MMSE, UPDRS motor score, dyskinesia assessment, and medication lists. At baseline vitamin D, calcium, and balance measures will be obtained. Normocalcaemic patients with vitamin D levels between 20ng/ml and 40ng/ml will then be randomized based on vitamin D levels and balance performance (SOT composite score). They will be assigned to one of two groups: (1) placebo plus 1000mg calcium or (2) 10,000 IU vitamin D plus 1000mg calcium once daily for 5 days each week. Due to the variation in the production of vitamin D, each 10,000 IU capsule may actually contain up to 15,000 IU of vitamin D and patients will be asked to take a maximum of one capsule daily. Vitamin D, ionized calcium, phosphate, and creatinine levels will be checked every 4 weeks and reviewed by Dr. Chaim Vanek, a board-certified endocrinologist, to ensure safety of this high dosage. At 16 weeks of supplementation subjects will undergo repeat balance testing. We did not feel it was ethical to enroll persons with vitamin D insufficiency (<20 ng/ml) into the placebo dose arm; but we did not want to lose the data from this population as these subjects might have the most dramatic response to Vitamin D supplementation. Therefore those with levels less then 20ng/ml (insufficient) will be enrolled at the high dose (10,000 IU/day for 5 days a week) level for 16 weeks in an open label fashion. Subjects with abnormalities in other lab values will not be enrolled in the study and will be referred to their PCP for management.

**Study Design**

Vit D

level

Not enrolled

Vit D & Ca++ levels

**Baseline**

**Testing**

>40

RANDOMIZE

**High dose vit D & Ca++**

Reassessment

20-40

Reassessment

Placebo & Ca++

High dose vit D & Ca++ (open label)

<20

Reassessment deleted

4 8 12 16

Weeks

**Figure 8:** shows the study design. Starting with baseline testing, randomization based on vitamin D levels, high or placebo dose supplementation, and repeat testing at 16 weeks. Subjects with vitamin D less than 20 ng/ml will be treated in an open-label fashion with high dose supplementation.

Subjects will be recruited from clinics at the Portland VA and OHSU, and from the community. Subjects who agree to participate will come to the Oregon Clinical and Translational Research Institute (OCTRI) on the 10^th^ floor of the Hatfield Research Center at OHSU. Subjects will be consented in one of the exam rooms by the PI, Dr. Amie Hiller. The study coordinator will then perform the iTUG. Labs will then be drawn across the hall by the OCTRI staff for vitamin D, ionized calcium, phosphate, and creatinine. The Principal Investigator will perform a neurological examination, past medical history, and the MMSE. The subjects will then be walked down to the 8^th^ floor rehab department where a physical therapist or Principal Investigator will perform Parkinson’s disease assessment, dyskinetic and balance assessments. The subjects will tehn be taken down to the Center for Health and Healing (CHH) and have strength testing performed. The subjects will complete a falls diary for the next four weeks prior to enrollment. If the subjects meet inclusion criteria they will be randomized at visit 1a and have further cognitive testing, and complete questionnaires. At visit 1a, subjects will be randomized, falls diary will be distributed and reviewed and the first month of study drug will be dispensed. Visits 2, 3, and 4 will also take place at OCTRI (either in the main hospital or at CHH dependent on subject and provider preference). Again they will have blood drawn for vitamin D, ionized calcium, phosphate, and creatinine. At visits 2, 3, and 4 pill counts will be done, falls diaries reviewed, the next 4 weeks of medications given, and labs repeated. Dr. Vanek will review the labs after each visit and inform the study coordinator if any patients need to be removed from the study. The 5^th^ and final study visit is similar to the first visit and will take place at OCTRI in the main hospital, the rehab department on the 8^th^ floor of the main hospital building, and balance testing in rehab at CHH. Subjects will undergo the full battery of PD severity, medical history, cognitive, depression, quality of life, laboratory, balance, dyskinesia, and strength testing. Patients will be asked to keep falls diary for the 8 weeks following the study and mail them in. They will be contacted by phone 8 weeks after completion and asked about any subsequent potential side effects from the intervention during the study.

**D.2 Study Population**

Inclusion Criteria: (1) Medically confirmed diagnosis of PD by a movement disorders specialist using the National Institute of Neurological disorders and Stroke (NINDS) criteria. (2) Ability to ambulate 50 feet without the assistance of another person. (3) Ability to cooperate with balance testing. (4) 50 + years of age. (5) Vitamin D level between 20 ng/ml and 40ng/ml. (6) Some degree of balance dysfunction indicated by: a score of 1 or greater on the pull test or 1 fall in last month or 2 near falls in the last month.

We have chosen to focus on person with at least some minor balance problems, a medical history of at least two near falls within the last month or some degree of balance dysfunction as measured by the pull test score of ≥ 1. People with very severe balance deficits would not be able to complete testing so subjects need to be able to walk at least 50 feet without the assistance of another person. We have chosen persons with insufficiency and low sufficient levels of vitamin D assuming that persons with lower levels will respond more robustly to supplementation, as has been seen in other studies.

Exclusion Criteria: (1) Significant cognitive deficits as defined by a Mini Mental Status Exam (MMSE) of <25. (2) Another neurological or orthopedic deficit that in the investigator’s opinion would have a significant impact on gait and cognition (e.g. stroke, fracture). (3) History of renal stones or renal disease (history of renal transplant, currently on dialysis, or a creatinine > 1.5 at baseline testing). (4) Subjects cannot be on > 600 IU vitamin D supplementation during the study. (5) Hypercalcaemia (based on ionized calcium level). (6) Known tuberculosis infection who have not been treated. (7) Pregnancy. (8) Soy Allergy.

For safety we will exclude anyone with hypercalcaemia(or a history of this), a history of kidney stones, renal disease, or untreated tuberculosis as the risk of the risk of hypercalcaemia and stones are increased with supplementation in these particular populations. Since we are using a fairly high dose for the vitamin D arm, we have also added the additional measures of phosphate and creatinine. As some other medical conditions such as a severe stroke or major orthopedic problem could affect testing, the PI will have discretion to not enroll such persons. We did not want to have frankly demented patients as cognitive testing could affect ability to cooperate with testing.

**Table 4: Detailed time line**

| **Year of grant (date)** | **Activities** | **# of subjects completed** |
| --- | --- | --- |
| 1 (July 2010-2011) | IRB approval  Animal research project  Recruitment  Begin data collection | 40 |
| 2 (July2011-2012) | Data collection | 80 |
| 3 (July 2012-2013) | Data collection | 120 |
| 4 (July 2013-2014) | Data collection  Analysis of baseline data | 140 |
| 5 (July 2014-2015) | Data analysis  Manuscript Preparation | 140 |

Randomization: We will use a permuted block randomization scheme to allocate treatments using strata determined by two factors: baseline vitamin D levels (>20-<30 vs. >30-<40ng/ml) and baseline composite SOT4-6 ( <64 vs. >64). This is based on what is considered insufficiency of vitamin D and the mean normative SOT score for persons 70-79 years old respectively.^59, 92^ Those with < 20 ng/ml baseline vitamin D levels will be enrolled into the vitamin D supplementation arm and will receive 10,000 IU of vitamin D for 16 weeks in an open label fashion. Those with > 40 ng/ml at baseline will not be enrolled in this study.

**D.3 Study Procedures**

D.3.1 Balance Measures: To limit variability in PD symptoms and medication effects throughout the day assessments will occur at the same time for each individual subject. This will be approximately half way between PD medication doses to attempt to get subjects in the “on” state. This is when most falls occur and how most patients try to spend the majority of their waking hours. The primary outcome measure will be a composite score of static and dynamic balance, as measured by the SOT composite score using dynamic posturography (see D.5.1). SOT composite includes 6 scenarios that replicate daily activities that often lead to falls. As shown by our preliminary data it is sensitive to balance problems seen in PD and has been shown to correlate with falls in other populations.^90^ The SOT was chosen from a much larger array of balance tests available based on the pilot study data (see C.4.1). Subjects performed moderately on this test such that there should not be a ceiling affect (where improvement could not be seen) or a floor affect (where subjects perform so poorly that even modest improvement would not be detectable). In addition if the SOT composite is not sensitive to balance changes we will calculate the root mean square velocity in the medial lateral direction (see C.4.2). Turning duration is also included for an ambulatory balance measure. Freezing of gait is a major cause of falls in PD and would not be evident with the SOT.^25^ This also has been shown sensitive to balance problems in PD and able to detecting balance problems early in PD when traditional testing in negative.^30^ This will be recorded using the iMOBILITY device and an instrumented timed up and go test (see D.5.2).

**Table 5: Time Table and Organizational Chart**

| Prior to study visits: | | Identification of potential subjects | | |
| --- | --- | --- | --- | --- |
| **Study Visit 1**  Screening Visit  Main Hospital  (120 minutes) | Review of inclusion & exclusion criteria  Consent  (5 minutes) | **Measures:**  (75 minutes)  Balance: SOT,MCT, iTUG | **Laboratory:**  (15 minutes)  Vitamin D  Ionized Ca^++^  Phos  Creatinine  Serum beta hCG | **Possible Confounder:**  (30 minutes)  Medication list  Cognitive tests (MMSE)  PD severity(UPRDS)  Dyskinesia |
| **Study Visit 1 a**  Enrollment  CHH  (90 minutes) | Randomization | **Measures:**  (20 minutes)  Strength: Biodex  QOL: PDQ39, NHP, ABC, POMS, Falls diary review |  | **Possible Confounder:**  (70 minutes)  Other cognitive testing  Medication List |
| **Study Visits**  **2-4**  Main Hospital or CHH  (30 minutes) | Review of lab inclusion & exclusion criteria | **Measures:**  (15 minutes)  Falls diary review  Pill count | **Laboratory:**  (15 minutes)  Vitamin D  Ionized Ca^++^  Phos  Creatinine | Distribution of Study Drugs |
| **After Visits 2-4** | Review of laboratory data | Telephone contact with patient and withdraw from study if elevated vitamin D, Ca^++^, Phos, creatinine | | |
| **Study Visit 5**  Main Hospital and CHH  (150 minutes) |  | **Measures:**  (75 minutes)  Balance: SOT, MCT, iTUG  Strength: Biodex  QOL: PDQ39, NHP, ABC, POMS | **Laboratory:**  (15 minutes)  Vitamin D  Ionized Ca^++^  Phos  Creatinine | **Possible Confounder:**  (30 minutes)  Medication list  Cognitive tests  PD severity(UPDRS)  Dyskinesia |
| **Phone Call**  **(10 minutes)** | Review of any potential adverse events | **Falls diary (mailed)** |  |  |

**D.3.2 Strength measures**

The frequency that a person falls is known to be affected by muscle strength.^93^ Severe vitamin D deficiency can cause myopathy and studies show that vitamin D supplementation improves muscle strength.^61^ In the proposed studies we will measure strength of leg flexion and extension. These are the major muscles involved in gait and are fairly easy to test using the Biodex system. We chose Biodex computerized dynamometer testing because this allows for more sensitive and consistent data then hand held dynamometers and can calculate total work as well as peak torque, a better measure of muscle function.^94^ We will use total work in the analysis. The baseline data in and of itself will be of interest as there is little data about strength changes in persons with PD.^95, 96^

**D.3.3 Quality of Life Measures**

Because falls and mobility issues play a major role in quality of life for persons with PD we expect to see these measures improve if there is a clinically meaningful change in balance.^17, 97^ We chose 2 scales, the NHP being a general QOL measure and the PDQ-39 a PD specific scale.^98, 99^ Study participants will be asked to complete the NHP and the PDQ-39 at visits 1 and 5. Both questionnaires generate a score of 0 to 100. The NHP asks 38 yes or no questions in categories of emotional reactions, sleep, energy, pain, physical mobility, and social isolation. The PDQ-39 has 39 items in categories of mobility, activities of daily living, emotional well-being, stigma, social support, cognitions, communication, and bodily discomfort. Both scales have been validated for use in PD.^99^ Because fear of falling also results in limitation we will perform the 16 item Activities-specific Balance Confidence (ABC) Scale at visits 1 and 5.^15^ The ABC scale questions percent confidence (0-100%) subjects have for not losing their balance during a variety of activities. Depression will be measured using the profile of Mood States (POMS) questionnaire, a 30-item mood questionnaire.

**D.3.4 Intervention**

Subjects will be assigned to one of two groups: (1) placebo plus 1000 mg calcium or (2) 10,000 IU vitamin D up to a maximum of 15,000 IU plus 1000 mg calcium once daily for 5 days each week. Vitamin D, calcium, phosphate, and creatinine levels will be checked every 4 weeks to be sure patients’ levels are not too high. With new locations and functions of vitamin D being discovered definition of sufficient and insufficient levels are in question. The recommended daily allowance (RDA) of vitamin D for persons 51-70 years old is 400 IU and for those over 71 years old is 600 IU.^100^ We have chosen a fairly aggressive supplementation with the rational that in a short term study getting higher levels sooner would increase the chance of seeing a change. Since the study is of such short duration and we are not including persons with clear deficiency we feel a true placebo arm is justified and will make the study stronger.

A weekly dose of 50,000 IU to 75,000 IU is felt to be a safe dosage. Other studies are approved using levels over 7,000 IU a day for an entire year. Data is limited in humans but levels consistently above 200ng/ml are considered potentially toxic.^62^ Even the 200ng/ml level may be safe and more in-depth animal studies have not indicated toxicity in levels under 400ng/ml.^101^ We will check levels monthly and for the utmost safety, subjects will be removed from the study if their levels are above 100ng/ml. In addition since the most common problem with vitamin D supplementation is hypercalcaemia, we will check monthly ionized calcium as well as phosphate, and creatinine for added safety.

The vitamin D will be obtained from Capsugel and the calcium carbonate directly from the VA pharmacy (BTR Group Inc is source). The regiments are detailed in the table below. Each subject will take pill 5 days a week (Monday thru Friday).. The vitamin D placebo is also manufactured by Capsugel and has an identical appearance to the active capsule . Both will be provided in bottles to assist with compliance and ease of administration and pill counting.

**Table 6: Medication Regiment for Placebo and High Dose Groups**

| **Placebo Group (M-F)** | **High Dose Group (M-F)** |
| --- | --- |
| 2-Calcium 500mg  1-Placebo (blinded) | 2-Calcium 500mg  1 Vitamin D3 10,000 IU up to a maximum of 15,000 IU (blinded) |

**D.3.5 Falls Assessment**

Subjects will be given a diary in which to record their falls at visit 1. They will be instructed to write the time of day of the fall, what they were doing when they fell, if they sustained an injury, and classify it as a fall or near fall. Falls will be defined to subjects as unintentionally coming to rest on the ground, or another lower level such as a chair, toilet, or bed.^102^ A near-fall will be defined as a slip (sliding of the support leg), trip (impact of the swinging leg with an external object) or loss of balance where the person starts to fall but is able to stop or prevent the fall to the ground or other lower surface.^103^ Subjects will be asked in person every 4 weeks when they meet with the study coordinator how often they fell and the diary will be reviewed. They will also be asked to keep a falls diary for the 8 weeks after study completion and return these by mail. Even though the study is not powered for falls and 16 weeks is a very short period to measure changes in fall frequency we felt it was important to collect this data.

**D.3.6 Monthly Labs**

In addition to the baseline measures, blood will be drawn every 4 weeks to serum check 25-hydroxyvitamin D (25-OHD), ionized calcium, phosphate, and creatinine after supplementation is begun. This is more extensive and frequent testing than generally done with supplementation, but because of the high dose arm we want to ensure absolute safety. Labs will be reviewed by Dr. Vanek. If a marked elevation or decrease of vitamin D levels (>100 ng/ml), ionized calcium (>1.32 mmol/L), phosphate (> 5.5 mg/dL), or creatinine (>30% change) are found, the subjects will perform the final study visit and will discontinue in the study. This is a safety measure and not part of the inclusion/exclusion criteria. 15 mL of blood from the screening visit and the final visit will be banked in a VA approved bio-repository.

**D.3.7 Possible Confounders**

Balance and falls in PD are known to be affected by many factors. Certain medications, such as benzodiazepines, can worsen balance in PD patients.^93^ Depending on the point in their dosing cycle PD severity and dyskinesias (dance-like abnormal involuntary movements) can fluctuate and affect balance. Cognition and a person’s level of physical activity could change over the course of a 4 months study and affect their likelihood of falling.^104^ Therefore, at the baseline and reassessment visit, Dr. Hiller will administer and record the following assessments:

(1) A medical history: This is to identify the subject’s current medications (subjects will be asked to bring in prescription bottles to every visit) and any new medical issues that could have a significant impact on balance testing (i.e. fracture, stroke). Subjects will be asked to not change any medications during the course of the 16 week study. Specifically patients will be instructed not to start or change doses of amantadine, cholinesterase inhibitors, antidepressants, or benzodiazepines as these may all have significant effects on balance.

(2)PD assessment: The Unified Parkinson's Disease Rating Scale (UPDRS) motor score, a very widely used 30 point scale of severity of PD physical symptoms will be recorded.^105^

(3) Dyskinesia Assessment: This will be measured via a clinical rating scale, the Modified Abnormal Involuntary Movements Score (mAIMS), and an objective measure, the force place dyskinesia rating (see D.5.4).^106, 107^

(4) Cognitive testing: The mini-mental status exam (MMSE) will be used for inclusions and the more in-depth Montreal Cognitive Assessment (MoCA), which has been shown to be sensitive to cognitive deficit in PD, will be used to follow cognitive function at the first and last visit.^109,^ ^110^ In addition the following cognitive tests will be administered: Trail Making A & B, Digit Symbol (WAIS-R), Judgment of Line Orientation, Stroop, Letter-Number Sequencing (WAIS-III), semantic fluency “animals” and “vegetables”, Logical Memory I and II (WMS-R), Boston Naming Test, and Digit Span (WAIS-R).

**D.4.0 Statistical Analysis**

D.4.1 Sample Size: We are basing our power calculation on the primary outcome, the composite SOT (cSOT) score from computerized posturography, for Aim 1. Although we are not assessing power for other outcomes, we will have a reasonable amount of data, targeting 70 subjects per group, to obtain useful information about additional outcomes. Using preliminary data on 20 subjects (see C.4), the average cSOT (SD), excluding one score of zero, was 58.5 (19.1). For conducting a power calculation on the cSOT measure we assume that 10% of subjects will score zero at baseline and follow-up. This is conservative since only 5% of the 20 subjects used for our preliminary data scored zero at a single time point. Among the remaining subjects, we assume SD’s of 19.1 at baseline and follow-up, that the high dose group will have an average score that is 8.8 points higher than at baseline (15% change from 58.5) and the placebo group shows no improvement. There are not studies looking at changes in SOT with vitamin D supplementation, but the Pfeifer did look at body sway, finding a 15% greater improvement in body sway in the intervention versus the control group.^111^  With 10% zeros this 15% change among the non-zeros translates to a mean difference in change of 7.9 points. The SD of the changes from baseline depends on the SD’s at baseline and follow-up and on the correlation between baseline and follow-up measures. We hence obtained sample size requirements using a range of correlation coefficients.

Using simulation with PASS 2008 software, we determined (see Table 7), the sample sizes needed to detect the 7.9 point mean difference indicated above with 80% power at significance level 0.049 (allowing for an interim analysis at significance level .001) for correlations between 0.5 and 0.75. The simulation method was based on the Mann-Whitney nonparametric test which is more conservative in this setting than a t-test procedure (that is, using a simple t-test for the simulation would result in smaller required sample sizes). We will adjust for confounders using parametric methods, but used the non-parametric approach for sample size selection to assure that enough subjects are selected for simple comparisons. The Balance Manager Systems Clinical Interpretation Guide for Computerized Dynamic Posturography report test-retest reliability results ranging from 0.66 to 0.98 for the cSOT.^92^ With 0.66 for correlation, we would require 63 subjects per group. Allowing for 10% drop-out we will then plan to recruit 70 subjects per group.

**Table 7. Sample sizes per group needed for 80% power at significance level 0.049 to detect a mean difference in posturography composite change of 7.9.**

| SD at baseline and at follow-up for composite posturography | Correlation between baseline and follow-up composite posturography | SD of difference between follow-up and baseline scores | Sample size per group |
| --- | --- | --- | --- |
| 19.1 | 0.50 | 19.1 | 91 |
| 19.1 | 0.66 | 15.8 | 63 |
| 19.1 | 0.75 | 13.5 | 47 |

D.4.2 Interim Analysis: When half of the targeted subjects have completed the follow-up visit, we will conduct an interim analysis of the primary outcome using a significance level of 0.001. This was felt appropriate to ensure additional safety for patients. The primary outcome analysis and analysis of other outcomes will be presented to the Data Safety Monitoring Committee, which will be made up of a group of researchers not directly involved in this project. Using a small significance level for the interim analysis requires strong evidence of an effect of Vitamin D for consideration of early stopping (We will use a significance level of 0.049 for the final analysis so that the Type I error rate for the primary outcome remains controlled at 0.05.). The details of the methods used at the interim and/or final analyses are described below.

D.4.3.1 Objective 1 Analysis: The primary analysis will use a Mann-Whitney test to compare the changes from baseline in the cSOT between the placebo and vitamin D groups. We will then use regression methods to adjust for the factors used in randomization, baseline vitamin D level and baseline cSOT, as well as the most important confounders: severity of  motor symptoms of PD (UPDRS motor score) and dyskinesia (clinical dyskinesia scale). With 140 subjects we have more than adequate data to adjust for 4 covariates. Additional covariates that are found to be statistically significant when added to the above model or alter the treatment effect estimate by more than 10% will be included in a second model. In addition to formal tests to compare treatment groups, we will construct 95% confidence intervals for the changes in mean cSOT within each treatment group and for the difference in mean cSOT between treatment groups.

Since Vitamin D serum levels depend on additional factors besides supplementation dose including dietary and sun exposure as well as compliance with study treatment we will perform secondary analyses in which we incorporate serum vitamin D level changes as a covariate in the model allowing us to examine the relationship between cSOT changes and vitamin D level changes.

The use of regression methods assume that residuals follow an approximately normal distribution. If the data do not support this assumption we will consider transformations of the response. If we have more than a few cSOT of zero, we will do a separate analysis of the non-zero scores to determine how this affects our results. We only observed a single cSOT of zero in 20 subjects in our preliminary data, so that we are not anticipating that this will be a significant issue in our study. Also, since we are comparing change from baseline, outliers (other than zero) at a single time point are not likely to result in extreme outliers among the change scores. Hence, other values (besides zero) that are extreme are not expected to have much impact on our results. We will, however, examine the influence of all data points (including outliers) on our conclusions.

We have increased our sample size to accommodate 10% drop-out in our power calculations. However, if subjects dropping out are different from those that do not drop out this introduces bias in our conclusions. If there is more than negligible drop-out we will conduct sensitivity analysis to assess the impact on our results of various assumptions about the reasons for drop-out. Information from participants will be solicited about the reasons for dropping-out to help inform our sensitivity analysis.

The secondary outcome for objective 1 will be analyzed in a similar fashion. For this outcome, root mean square velocity, we will not have the issue of zero scores as we did for the composite posturography measure. The measure of dynamic balance, turn velocity, will also not have the issue of zeros.

D.4.3.2 Objective 2 Analysis: In order to determine if changes in balance are due primarily to changes in strength, we will use regression methods. First we will do analyses similar to above for the static posturography measures (SOT1-SOT3), dynamic posturography measures (SOT4-SOT6), and turn velocity from the iMOBILITY. For the individual dynamic measures, scores of zero will occur more commonly than with the overall composite score and hence we will first compare the proportion of individuals who score zero at baseline, follow-up, and both at baseline and follow-up between the two treatment groups. Descriptive information will be obtained on each of the scores. We will again use the Mann-Whitney test to compare the changes from baseline for each component score between the groups. Composite dynamic and static scores will be obtained by averaging the component scores. The dynamic and static composite scores will be compared using the Mann Whitney test and with regression to adjust for confounders. Again, we expect the composite scores to exhibit few zeros. The influence on our results of zeros (and other data values) will be examined.

We will then compare changes in strength between the two groups using regression methods. We will examine the relationship between changes in strength and changes in the dynamic balance composite score, the static balance composite score, and turn velocity using linear regression methods. The proportions of the variance in the changes in balance and turn duration measures that are explained by the changes in strength will be obtained. In addition to examining the relationship of changes in strength to changes in balance and turn velocity overall, we will examine the relationship after adjustment for treatment group and the confounding variables described above. As in aim 1, we will conduct a secondary analysis to relate changes in outcomes to changes in serum vitamin D.

D.4.3.3 Objective 3 Analysis: For aim 3, we will use the same approaches as above to compare quality of life measures between treatment groups the NHI and the PDQ-39. As a secondary measure to better understand the effect on fear of falling, we will compare the ABC scales between the 2 groups.

D.4.3.4. Additional Analysis: Even though not the primary aim of the study, we feel it would also be useful to look at changes in motor and cognitive function not measured by the gait and balance tests. Therefore we will analyze the UPDRS motor scores and cognitive measures with the same approach as for the other objectives.

**D.5 Detailed Methods**

D.5.1 Sensory Organization Test (SOT): This test is carried out using computerized posturography, a system with a moveable platform and surround that containing multiple force plates recording weight transfer (see Figure 9). Subjects are placed in a harness and stand on the platform. There are 6 different scenarios (see Figure 10). Scenarios 1-3 test static balance. The platform is stable and the subject is told to stand as still as they can first with eyes open, then closed, then open and the surround is in motion. Scenarios 4-6 test dynamic balance. The base moves in response to the subjects own motion, termed sway reference. Scenario 4 is with eyes open, scenario 5 is with eyes closed, and scenario 6 is with the surround moving. Figure 11 shows the results from one of the pilot subjects. Each scenario has 3 trials. Weight movement in the anterior posterior direction is rated on a score of 0 fall) up to 100. The change in a composite score of the 6 scenarios will be used as the primary outcome measure.

**Figure 9: Computerized Posturography**

**Figure 10: SOT Scenarios**

**Figure 9:** shows a subject in the harness on the computerized posturography system.


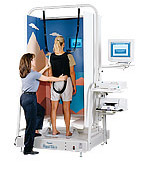


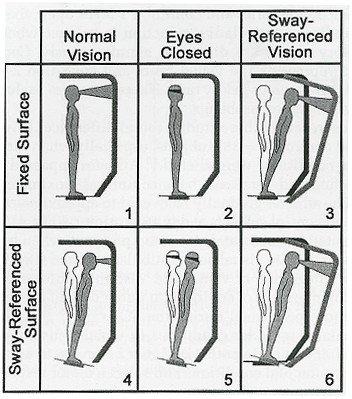


**Figure 10:** shows the 6 SOT scenarios

**Sample Sensory Organization Test**

**Figure 11:** shows the SOT results for a pilot study subject. He does well in scenarios 1, 2, and 4; but has frequent falls in scenarios 5 and 6 and a low composite score. The light bars are considered within normal limits for age (5^th^ percentile) and the dark bars are worse than this.

D.5.1b Motor Control Test (MCT): This test will also be carried out using computerized posturography. The MCT assess the ability of the automatic motor system to quickly recover following an unexpected external disturbance. Sequences of small, medium, or large platform translations in forward and backward directions elicit automatic postural responses.

D.5.2 iMOBILITY: Figure 12 shows the iMOBILITY device alone and worn by a subject. We will be using this device during a timed up and go (TUG) in which the subject sits in a chair and when told to start rises, walks to a line 3 feet away, turns, and returns to a seated position. The sensors are interfaced with a computer and able to record over 50 metrics (many more than for a tradition TUG) including, arm swing, asymmetry, number of steps, and turn duration. Recent studies show that iMOBILITY has excellent test-retest reliability, very good concurrent validity with traditional rating scales of balance and gait and high sensitivity to exercise intervention of iMOBILITY for patients with PD.^30,^ ^87^ This test is able to detect changes in early PD when traditional stop watch TUG is within normal limits. We specifically plan to use the turn duration which has been shown to change with disease progression and correlate to with clinical measures of disease severity (see C.4.3).

D.5.3 Strength Measures: Subjects will be lead through strength testing using the Biodex machine (see Figure 13). The subjects are seated in the Biodex and one leg is placed in the machine. Subjects are encouraged to flex and extend their leg for 5 repetitions as fast and as hard as they can. This is then repeated on the other side. The Biodex is a computerized dynamometer that we will use in an isometric setting set at 120 range of motion. It gives a constant degree of resistance and is able to record total work and peak torque. This device is more sensitive and has better test, re-test reliability than traditional hand held dynamometers.^88,^  ^112^ The baseline data in and of itself will be very interesting. Persons with PD likely have lower extremities that are weaker than age matched controls, but this rarely has been looked at in a rigorous manner.^95^ Dr. Hiller has not used this equipment in a research protocol, but our physical therapists have used it clinically in patients with PD with good success.

**Figure 12: iMOBILITY sensors**

**Figure 13: Biodex Computerized Dynamometer**


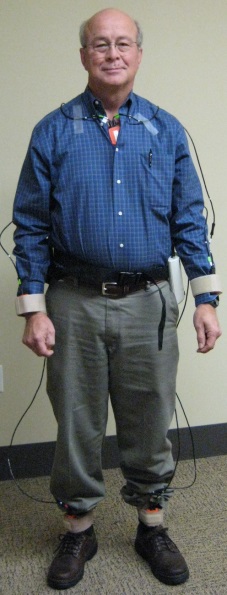

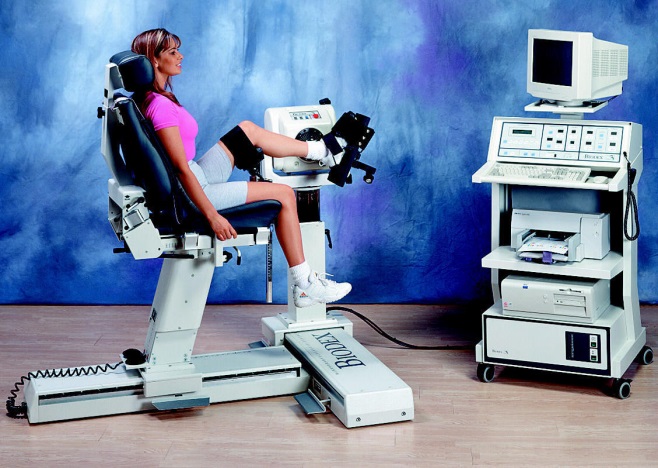


D.5.4 Force Plate Dyskinesia Rating: Dyskinesias can be problematic in PD and can be particularly problematic using a devise that measures how much movement a person experiences. The force plate may detect movement and record the individual as having poor balance, when in fact the movements are dyskinesia rather than postural adjustments. Like the UPDRS motor score, dyskinesia scores can fluctuate throughout the day. In addition to a subjective measure of dyskinesias, the Modified Abnormal Involuntary Movements Score (mAIMS), we will use an objective measure, the force plate dyskinesia rating. We will follow a protocol designed by Dr. Kathy Chung, also at OHSU.^107^ Subjects with stand on the computerized posturography platform for 50 seconds with arms at their sides, on a steady base, and asked to name animals. The root mean square of the velocity (RMSV) in the anterior posterior direction will be calculated. Dr. Chung’s research shows this measure correlates well with the subjective mAIMS, but displays greater sensitivity (see Figure 15).

**D.6 Possible Pitfalls:**

Recruitment is not expected to be an impediment. Our center is historically very successful in recruiting patients and the inclusion criteria here are quite broad. With a study coordinator involved the pilot study enrolled 8-10 subjects a month, with many more expressing interest. Retention of subjects for 16 weeks in study of a treatment (high dose vitamin D) which is available outside of the study may be challenging. We will also provide $10 per visit and the statistical analysis plan accounts for a drop-out rate of 10%. There are other sources of vitamin D including sun exposure and diet. By choosing a fairly high dose for the intervention group dietary and sun sources are likely to be minimal in comparison. In addition by measuring plasma vitamin D levels we can identify subjects who “drop in” with surreptitious or inadvertent use of vitamin D supplements, dietary changes, or changes in sun exposure related to season. Eight or 12 weeks may be an insufficient treatment period over which to see a treatment effect—this is precisely why outcomes are measured at 16 weeks.

**Figure 15: Sensitivity of RMSV to Measure Dyskinesias**

**Time of measure**

IV Levodopa Infusion

- RMSV
- mAIMS

**U**

**n**

**i**

**t**

**V**

**a**

**l**

**u**

**e**

**Figure 15:** shows that the root mean square of the velocity (RMSV) is more sensitive than a clinical scale to detect dyskinesias in PD patients with levo-dopa induced dyskinesias

It may be that the sensory organization test (SOT), timed up and go (TUG), and handheld dynamometers are not sensitive enough. Because we have one of only 8 research level dynamic posturography systems in the world we are able to look at more sensitive measures of balance using the data by calculating the root mean square velocity in the medial lateral direction. iMOBILITY allows for much more sensitive measures than a traditional TUG. Changes in strength are often somewhat difficult to assess with a hand held dynamometer, which are notoriously inconsistent. Using the Biodex machine the subject is aligned in a similar matter with each testing session and results are more consistent.^88, 112^

In reviewing clinicaltrials.gov, there are 2 other studies looking at PD and vitamin D. Our study is unique in the in-depth measures of balance and strength utilized. We will be able to comment on the contribution of strength, see if vitamin D is beneficial, and see what aspects of gait and balance are influenced. In reviewing more broadly studies on vitamin D and falls or balance in other populations on clinicaltrials.gov, none have included clinical posturography or similarly sensitive measures as outcomes. The in-depth data obtained in this study and the focus on the distinction between strength and central nervous system affects on balance is a first step to better understanding if and how vitamin D reduces falls in PD and other populations.

**D.7 Future Steps**

This study is important in learning about vitamin D and balance in PD. If vitamin D is effective clarifying the optimum levels and looking at system based changes to make vitamin D assessments a routine part of comprehensive PD care would be appropriate. Even if vitamin D is not beneficial, understanding balance and strength in PD and the experience with computerized posturography, iMOBILITY, and Biodex is important for Dr. Hiller to develop expertise with state of the art tools. As other symptoms of PD are better managed by medications and surgical interventions and our population ages, postural instability is a major concern and a major focus of research in PD. Dr. Hiller’s training with this grant and the resources available both in terms of personnel and equipment at the Portland VA and OHSU, will position her to successfully transition to an independent researcher and position the Portland VA as a leader in an area with great need.

**E. Human Subjects**

**E.1 Potential Risks and Protection from Risks**: There is minimal risk involved in the proposed research. The blood draws may result in slight pain, bleeding, bruising, or infection. To decrease the likelihood of such complications, all blood draws will be performed by trained phlebotomist. There is a risk that subjects may fall during balance tests, therefore the most difficult testing will take place with them wearing a safety harness. This is a device that wraps around the subjects’ shoulders, waist, and legs and is attached to the equipment (see D.5.1). In addition for all the testing a technician will be standing within arm’s length of the subject. The most concerning potential complication from vitamin D supplementation is hypercalcaemia. Persons with hypercalcaemia or a history of this will be excluded, as will persons with a history or kidney stones. Ionized calcium, vitamin D, phosphate, and creatinine levels will be checked prior to the start of the study and monthly thereafter to ensure complete safety. An interim analysis will be performed since there is limited data to predict out sample size. This safe guard will ensure that fewer subjects will be exposed to placebo treatment if high dose is clearly more beneficial.

For each visit occurring at Oregon Health & Science University, a member of the study staff and/or principal investigator will hand carry the paper chart from the VA Portland Health Care System to Oregon Health & Science University. The chart will remain under control of study staff at all times and will be hand carried back to the Portland VA at the end of the study visit. If the information that is either carried to OHSU or collected at OHSU is lost, the participant will be informed. Additionally, the VA information security officer will be informed and a serious adverse event will be reported to the OHSU IRB, and the VA IRB.

**E.3 Recruitment:** Clinicians and research coordinators will identify potential subjects from the pilot study and the movement disorders clinics at OHSU and the Portland VA. Monthly newsletters distributed by the PADRECC, Parkinson’s Center of Oregon (PCO) at OHSU, and the Parkinson’s Resources of Oregon (with circulations of 1,300; 5,000; and 5,000 respectively) will describe the study. Additionally flyers will be hung at OHSU and the VA. The PADRECC and PCO saw 480 and 585 patients with PD respectively in the last year. These numbers tend to rise every year. In addition, this does not include additional research subjects who are not seen clinically. A recent study on falls recruited 105 subjects with PD in just 18 months.

We intend to enroll 40 subjects a year. Planning on 10 subjects a month for 4 months, would result in 40 completing the 16 week study by the end of the 1^st^ year. Since receiving a study coordinator the pilot study has enrolled 8-10 subjects a month, with many others expressing interest. The inclusion criteria are fairly broad. The most restrictive factor is likely the vitamin D levels. Data indicates that it is likely about 20% (23% in Evatt study, 21% in MrOS data, 39% in Sato) of potential subjects will have vitamin D levels less than 20ng/ml.^4, 78,^ ^113^ More are likely to be in the 20-30ng/ml range (32% in Evatt, 50% in MrOS, 50% in Sato). The 30-40ng/ml range is harder to predict as this has not been reported in most studies. The mean serum vitamin D level however for the available studies at 31.9ng/ml, 25.3ng/ml, and 11.9ng/ml suggest that few subjects will have particularly high levels.

**E.4 Informed Consent:** Dr. Hiller will inform persons who meet inclusion criteria and will invite them to sign a consent form before participating. She will assign each subject a number for anonymity. For scheduling purposes prior to consent, a waiver of informed consent will cover the scheduling of the screening visit. The information used to enroll is name, address, phone number, date of birth, and social security number.

**E.5 Confidentiality:** Each subject will be identified with a unique numerical identifier, which will be used for all testing. The data will be kept in a locked file cabinet in a locked office when not being used. Dr. Chaim Vanek in the endocrinology department will review the labs throughout the course of the study and inform the research coordinator if subjects need to be removed from the study.

**E.6 Potential Benefit of Proposed Research to the Subjects and Others:** All subjects participating will be informed of their vitamin D level at some point during the study. Vitamin D deficiency and insufficiency often goes unrecognized. On a recent review of VISN20 data, less than 8% of persons with PD had ever had a vitamin D level checked.^5^ The information obtained, seeing if high dose vitamin D improves balance in PD, is important for the subjects involved. Most persons with PD experience balance difficulties at some point in the course of the disease and the more data that is known the better their management will be when that time comes. It is also possible that an individual subjects testing will reveal a balance disorder of a different etiology that could result in additional treatment and improvement.

**E.7 Importance of the Knowledge to be Gained:** The NIH conference, “Vitamin D and Health in the 21^st^ Century: an Update,” identified determining the mechanism through which vitamin D decrease the risk of falls in elderly persons as a key research need.^56^ This is a primary objective of this study, to gain understanding into the effects of the muscular and central nervous systems on balance with vitamin D supplementation characterize what aspects of balance improve. Falls are a major issue for people with PD as well as many other veterans. Every year almost half the population over 65 years experiences a fall.^114^ If higher vitamin D levels improve balance, inexpensive supplementation may be able to decrease fall rates and their associated morbidity and mortality. This has the potential to both improve care and decrease the cost of care for these patients.

**F. Resources**

PD patients are seen in clinics at both OHSU and the VA Portland Health Care System (VAPORHCS). The two institutions are connected by a pedestrian bridge. The OHSU’s Parkinson’s Center of Oregon and the VAPORHCS’s Parkinson’s Disease Research, Education, and Clinical Center (PADRECC) combined serve over 1000 patients with PD each year. The clinics are staffed by six neurologists trained in movement disorders, supported by nurses, social workers, and physical therapists.

VAPORHCS’s clinic space will not be utilized for the research project. The research project will be supported by OHSU’s Oregon Clinical and Translational Research Institute (OCTRI) located in the Hatfield Research building, 10^th^ floor. All blood draws will be completed by OCTRI. Blood draws will be processed at VAPORHCS for vitamin D levels, creatinine, phosphate, ionized calcium, and beta hCG. Balance and strength testing equipment; including the computerized posturography equipment, the iMOBILITY dynamic balance system, and the Biodex computerized dynamometer; will be provided by the OHSU Rehabilitation Center utilizing their space in the main hospital building, 8^th^ floor, this is very close to the OCTRI space.

VAPORHCS’s PADRECC will provide office space for Dr. Amie Hiller, as well as neurologists Drs. Joseph Quinn and Kathy Chung. OHSU’s PCO provides office space for Neurologists Drs. Jay Nutt, Penny Hogarth, Matthew Brodsky, Jeff Kraakevik, supporting nursing staff, and research assistants.

The PCO and PACRECC offices are supplied with networked computers for each neurologist and staff member.

**G. Literature Citation**

1. Peterson AL, Nutt JG. Treatment of parkinson's disease with trophic factors. Neurotherapeutics 2008.

2. Peterson AL, Andrews MN, Quinn J, Nutt JG. Dopamine antagonist prescribing practices to persons with parkinson's disease. Neurology 2009 May;72(11(suppl 3)):A457.

3. Peterson AL, Andrews MN, Quinn JF, Nutt JG. Instituting a medication comment in an electronic ordering system to decrease the prescription of dopamine blockers to parkinson's patients. Mov.Disord. 2008 May;15(23-supple1):S310.

4. Peterson AL, Fink HA, Quinn J, Barret-Connor E, Shikany J, Orwoll E. Vitamin D levels in men with parkinson's disease. Mov Disord 2008;23(11):1635.

5. Peterson AL, Quinn J, Duckart J, Nutt JG. Vitamin D and bone density assessments are rarely obtained in veterans with parkinson's disease. Mov Disord 2009;24(suppl 1):S373.

6. Peterson AL. Patient management problem. Continuum Lifelong Learning Neurol 2010;16(1).

7. Hausdorff JM, Lertratanakul A, Cudkowicz ME, Peterson AL, Kaliton D, Goldberger AL. Dynamic markers of altered gait rhythm in amyotrophic lateral sclerosis. J Appl Physiol 2000 Jun;88(6):2045-53.

8. Jackson C, Gaugris S, Sen SS, Hosking D. The effect of cholecalciferol (vitamin D3) on the risk of fall and fracture: A meta-analysis. QJM 2007 Apr;100(4):185-92.

9. Bischoff-Ferrari HA, Dawson-Hughes B, Willett WC, Staehelin HB, Bazemore MG, Zee RY, Wong JB. Effect of vitamin D on falls: A meta-analysis.[see comment]. JAMA 2004 Apr 28;291(16):1999-2006.

10. Latham NK, Anderson CS, Reid IR. Effects of vitamin D supplementation on strength, physical performance, and falls in older persons: A systematic review. J Am Geriatr Soc 2003 Sep;51(9):1219-26.

11. O'Donnell S, Moher D, Thomas K, Hanley DA, Cranney A. Systematic review of the benefits and harms of calcitriol and alfacalcidol for fractures and falls. Journal of Bone & Mineral Metabolism 2008;26(6):531-42.

12. Richy F, Dukas L, Schacht E. Differential effects of D-hormone analogs and native vitamin D on the risk of falls: A comparative meta-analysis. Calcif Tissue Int 2008 Feb;82(2):102-7.

13. Cyr DG, Moore GF, Moller CG. Clinical application of computerized dynamic posturography. Entechnology 1988 Sep:36-47.

14. Pfeifer M, Begerow B, Minne HW. Vitamin D and muscle function. Osteoporosis Int 2002 Mar;13(3):187-94.

15. Powell LE, Myers AM. The activities-specific balance confidence (ABC) scale. Journals of Gerontology Series A-Biological Sciences & Medical Sciences 1995 Jan;50A(1):M28-34.

16. Hagell P, Whalley D, McKenna SP, Lindvall O. Health status measurement in parkinson's disease: Validity of the PDQ-39 and nottingham health profile. Movement Disorders 2003 Jul;18(7):773-83.

17. Forsaa EB, Larsen JP, Wentzel-Larsen T, Herlofson K, Alves G. Predictors and course of health-related quality of life in parkinson's disease. Movement Disorders 2008 Jul 30;23(10):1420-7.

18. Tillerson JL, Caudle WM, Reveron ME, Miller GW. Exercise induces behavioral recovery and attenuates neurochemical deficits in rodent models of parkinson's disease. Neuroscience 2003;119(3):899-911.

19. Delbaere K, Crombez G, Vanderstraeten G, Willems T, Cambier D. Fear-related avoidance of activities, falls and physical frailty. A prospective community-based cohort study. Age & Ageing 2004 Jul;33(4):368-73.

20. Deshpande N, Metter EJ, Lauretani F, Bandinelli S, Guralnik J, Ferrucci L. Activity restriction induced by fear of falling and objective and subjective measures of physical function: A prospective cohort study. J Am Geriatr Soc 2008 Apr;56(4):615-20.

21. Hunt SM, McKenna SP, McEwen J, Backett EM, Williams J, Papp E. A quantitative approach to perceived health status: A validation study. Journal of Epidemiology & Community Health 1980 Dec;34(4):281-6.

22. Eyles DW, Smith S, Kinobe R, Hewison M, McGrath JJ. Distribution of the vitamin D receptor and 1 alpha-hydroxylase in human brain. J Chem Neuroanat 2005 Jan;29(1):21-30.

23. Wood BH, Bilclough JA, Bowron A, Walker RW. Incidence and prediction of falls in parkinson's disease: A prospective multidisciplinary study. Journal of Neurology, Neurosurgery & Psychiatry 2002 Jun;72(6):721-5.

24. Bloem BR, van Vugt JP, Beckley DJ. Postural instability and falls in parkinson's disease. Adv Neurol 2001;87:209-23.

25. Bloem BR, Hausdorff JM, Visser JE, Giladi N. Falls and freezing of gait in parkinson's disease: A review of two interconnected, episodic phenomena. Movement Disorders 2004 Aug;19(8):871-84.

26. Stel VS, Smit JH, Pluijm SM, Lips P. Consequences of falling in older men and women and risk factors for health service use and functional decline. Age & Ageing 2004 Jan;33(1):58-65.

27. Gagnon N, Flint AJ, Naglie G, Devins GM. Affective correlates of fear of falling in elderly persons. American Journal of Geriatric Psychiatry 2005 Jan;13(1):7-14.

28. van Haastregt JC, Zijlstra GA, van Rossum E, van Eijk JT, Kempen GI. Feelings of anxiety and symptoms of depression in community-living older persons who avoid activity for fear of falling. American Journal of Geriatric Psychiatry 2008 Mar;16(3):186-93.

29. Wenning GK, Ebersbach G, Verny M, Chaudhuri KR, Jellinger K, McKee A, Poewe W, Litvan I. Progression of falls in postmortem-confirmed parkinsonian disorders. Movement Disorders 1999 Nov;14(6):947-50.

30. Zampieri C, Salarian A, Carlson-Kuhta P, Aminian K, Nutt JG, Horak FB. An instrumented timed up and go test characterizes gait and postural transitions in untreted parkinson's disease. J Neurol Neurosurg Psychiatry 2009 Sep 2.

31. Bloem BR, Beckley DJ, van Dijk JG, Zwinderman AH, Remler MP, Roos RA. Influence of dopaminergic medication on automatic postural responses and balance impairment in parkinson's disease. Movement Disorders 1996 Sep;11(5):509-21.

32. Beckley DJ, Panzer VP, Remler MP, Ilog LB, Bloem BR. Clinical correlates of motor performance during paced postural tasks in parkinson's disease. J Neurol Sci 1995 Oct;132(2):133-8.

33. Frank JS, Horak FB, Nutt J. Centrally initiated postural adjustments in parkinsonian patients on and off levodopa. J Neurophysiol 2000 Nov;84(5):2440-8.

34. Narabayashi H, Kondo T, Yokochi F, Nagatsu T. Clinical effects of L-threo-3,4-dihydroxyphenylserine in cases of parkinsonism and pure akinesia. Adv Neurol 1987;45:593-602.

35. Tohgi H, Abe T, Takahashi S. The effects of L-threo-3,4-dihydroxyphenylserine on the total norepinephrine and dopamine concentrations in the cerebrospinal fluid and freezing gait in parkinsonian patients. Journal of Neural Transmission - Parkinsons Disease & Dementia Section 1993;5(1):27-34.

36. Auriel E, Hausdorff JM, Herman T, Simon ES, Giladi N. Effects of methylphenidate on cognitive function and gait in patients with parkinson's disease: A pilot study. Clin Neuropharmacol 2006 Jan-Feb;29(1):15-7.

37. Devos D, Krystkowiak P, Clement F, Dujardin K, Cottencin O, Waucquier N, Ajebbar K, Thielemans B, Kroumova M, Duhamel A, Destee A, Bordet R, Defebvre L. Improvement of gait by chronic, high doses of methylphenidate in patients with advanced parkinson's disease. Journal of Neurology, Neurosurgery & Psychiatry 2007 May;78(5):470-5.

38. Hariz MI, Rehncrona S, Quinn NP, Speelman JD, Wensing C, Multicentre Advanced Parkinson's Disease Deep Brain Stimulation,Group. Multicenter study on deep brain stimulation in parkinson's disease: An independent assessment of reported adverse events at 4 years. Movement Disorders 2008 Feb 15;23(3):416-21.

39. Guehl D, Cuny E, Benazzouz A, Rougier A, Tison F, Machado S, Grabot D, Gross C, Bioulac B, Burbaud P. Side-effects of subthalamic stimulation in parkinson's disease: Clinical evolution and predictive factors. European Journal of Neurology 2006 Sep;13(9):963-71.

40. Weaver FM, Follett K, Stern M, Hur K, Harris C, Marks WJ,Jr, Rothlind J, Sagher O, Reda D, Moy CS, Pahwa R, Burchiel K, Hogarth P, Lai EC, Duda JE, Holloway K, Samii A, Horn S, Bronstein J, Stoner G, Heemskerk J, Huang GD, CSP 468 Study G. Bilateral deep brain stimulation vs best medical therapy for patients with advanced parkinson disease: A randomized controlled trial. JAMA 2009 Jan 7;301(1):63-73.

41. Deane KH, Jones D, Playford ED, Ben-Shlomo Y, Clarke CE. Physiotherapy for patients with parkinson's disease: A comparison of techniques. Cochrane Database of Systematic Reviews 2001(3):002817.

42. Keus SH, Bloem BR, Hendriks EJ, Bredero-Cohen AB, Munneke M, Practice Recommendations Development G. Evidence-based analysis of physical therapy in parkinson's disease with recommendations for practice and research. Movement Disorders 2007 quiz 600; Mar 15;22(4):451-60.

43. Goodwin VA, Richards SH, Taylor RS, Taylor AH, Campbell JL. The effectiveness of exercise interventions for people with parkinson's disease: A systematic review and meta-analysis. Movement Disorders 2008 Apr 15;23(5):631-40.

44. Kurtais Y, Kutlay S, Tur BS, Gok H, Akbostanci C. Does treadmill training improve lower-extremity tasks in parkinson disease? A randomized controlled trial. Clinical Journal of Sport Medicine 2008 May;18(3):289-91.

45. Hackney ME, Kantorovich S, Levin R, Earhart GM. Effects of tango on functional mobility in parkinson's disease: A preliminary study. Journal of Neurologic Physical Therapy 2007 Dec;31(4):173-9.

46. Hackney ME, Earhart GM. Tai chi improves balance and mobility in people with parkinson disease. Gait Posture 2008 Oct;28(3):456-60.

47. Horak FB, Frank J, Nutt J. Effects of dopamine on postural control in parkinsonian subjects: Scaling, set, and tone. J Neurophysiol 1996 Jun;75(6):2380-96.

48. Gallagher JC, Fowler SE, Detter JR, Sherman SS. Combination treatment with estrogen and calcitriol in the prevention of age-related bone loss. Journal of Clinical Endocrinology & Metabolism 2001 Aug;86(8):3618-28.

49. Bischoff HA, Stahelin HB, Dick W, Akos R, Knecht M, Salis C, Nebiker M, Theiler R, Pfeifer M, Begerow B, Lew RA, Conzelmann M. Effects of vitamin D and calcium supplementation on falls: A randomized controlled trial. Journal of Bone & Mineral Research 2003 Feb;18(2):343-51.

50. Grant AM, Avenell A, Campbell MK, McDonald AM, MacLennan GS, McPherson GC, Anderson FH, Cooper C, Francis RM, Donaldson C, Gillespie WJ, Robinson CM, Torgerson DJ, Wallace WA, RECORD Trial G. Oral vitamin D3 and calcium for secondary prevention of low-trauma fractures in elderly people (randomised evaluation of calcium or vitamin D, RECORD): A randomised placebo-controlled trial. Lancet 2005 May 7-13;365(9471):1621-8.

51. Flicker L, MacInnis RJ, Stein MS, Scherer SC, Mead KE, Nowson CA, Thomas J, Lowndes C, Hopper JL, Wark JD. Should older people in residential care receive vitamin D to prevent falls? results of a randomized trial. J Am Geriatr Soc 2005 Nov;53(11):1881-8.

52. Trivedi DP, Doll R, Khaw KT. Effect of four monthly oral vitamin D3 (cholecalciferol) supplementation on fractures and mortality in men and women living in the community: Randomised double blind controlled trial. BMJ 2003 Mar 1;326(7387):469.

53. Bischoff-Ferrari HA, Willett WC, Wong JB, Stuck AE, Staehelin HB, Orav EJ, Thoma A, Kiel DP, Henschkowski J. Prevention of nonvertebral fractures with oral vitamin D and dose dependency: A meta-analysis of randomized controlled trials. Arch Intern Med 2009 Mar 23;169(6):551-61.

54. Pfeifer M, Begerow B, Minne HW, Suppan K, Fahrleitner-Pammer A, Dobnig H. Effects of a long-term vitamin D and calcium supplementation on falls and parameters of muscle function in community-dwelling older individuals. Osteoporosis Int 2009 Feb;20(2):315-22.

55. Pfeifer M, Begerow B, Minne HW, Abrams C, Nachtigall D, Hansen C. Effects of a short-term vitamin D and calcium supplementation on body sway and secondary hyperparathyroidism in elderly women. Journal of Bone & Mineral Research 2000 Jun;15(6):1113-8.

56. Brannon PM, Yetley EA, Bailey RL, Picciano MF. Overview of the conference "vitamin D and health in the 21st century: An update". Am J Clin Nutr 2008 Aug;88(2):483S-90S.

57. Raiten DJ, Picciano MF. Vitamin D and health in the 21st century: Bone and beyond. executive summary. Am J Clin Nutr 2004 Dec;80(6 Suppl):1673S-7S.

58. Rajakumar K. Vitamin D, cod-liver oil, sunlight, and rickets: A historical perspective. Pediatrics 2003 Aug;112(2):e132-5.

59. Holick MF. Vitamin D deficiency. N Engl J Med 2007 Jul 19;357(3):266-81.

60. Thomas MK, Lloyd-Jones DM, Thadhani RI, Shaw AC, Deraska DJ, Kitch BT, Vamvakas EC, Dick IM, Prince RL, Finkelstein JS. Hypovitaminosis D in medical inpatients. N Engl J Med 1998 Mar 19;338(12):777-83.

61. Bischoff-Ferrari HA, Giovannucci E, Willett WC, Dietrich T, Dawson-Hughes B. Estimation of optimal serum concentrations of 25-hydroxyvitamin D for multiple health outcomes.[erratum appears in am J clin nutr. 2006 nov;84(5):1253 note: Dosage error in abstract]. Am J Clin Nutr 2006 Jul;84(1):18-28.

62. Jones G. Pharmacokinetics of vitamin D toxicity. Am J Clin Nutr 2008 Aug;88(2):582S-6S.

63. Heaney RP. Vitamin D: Criteria for safety and efficacy. Nutr Rev 2008 Oct;66(10 Suppl 2):S178-81.

64. Chesney RW. Vitamin D: Can an upper limit be defined? J Nutr 1989 Dec;119(12 Suppl):1825-8.

65. Shepard RM, DeLuca HF. Determination of vitamin D and its metabolites in plasma. Meth Enzymol 1980;67:393-413.

66. Bischoff-Ferrari HA, Conzelmann M, Stahelin HB, Dick W, Carpenter MG, Adkin AL, Theiler R, Pfeifer M, Allum JH. Is fall prevention by vitamin D mediated by a change in postural or dynamic balance?. Osteoporosis Int 2006;17(5):656-63.

67. Glerup H, Mikkelsen K, Poulsen L, Hass E, Overbeck S, Andersen H, Charles P, Eriksen EF. Hypovitaminosis D myopathy without biochemical signs of osteomalacic bone involvement. Calcif Tissue Int 2000 Jun;66(6):419-24.

68. Bischoff-Ferrari HA, Dietrich T, Orav EJ, Hu FB, Zhang Y, Karlson EW, Dawson-Hughes B. Higher 25-hydroxyvitamin D concentrations are associated with better lower-extremity function in both active and inactive persons aged > or =60 y. Am J Clin Nutr 2004 Sep;80(3):752-8.

69. Moreland JD, Richardson JA, Goldsmith CH, Clase CM. Muscle weakness and falls in older adults: A systematic review and meta-analysis. J Am Geriatr Soc 2004 Jul;52(7):1121-9.

70. Horlings CG, van Engelen BG, Allum JH, Bloem BR. A weak balance: The contribution of muscle weakness to postural instability and falls. Nature Clinical Practice Neurology 2008 Sep;4(9):504-15.

71. Lord SR, Allen GM, Williams P, Gandevia SC. Risk of falling: Predictors based on reduced strength in persons previously affected by polio. Archives of Physical Medicine & Rehabilitation 2002 Jun;83(6):757-63.

72. Lord SR, Clark RD, Webster IW. Postural stability and associated physiological factors in a population of aged persons. J Gerontol 1991 May;46(3):M69-76.

73. Topp R, Estes PK, Dayhoff N, Suhrheinrich J. Postural control and strength and mood among older adults. Applied Nursing Research 1997 Feb;10(1):11-8.

74. Carter ND, Khan KM, Mallinson A, Janssen PA, Heinonen A, Petit MA, McKay HA, Fall-Free BC Research G. Knee extension strength is a significant determinant of static and dynamic balance as well as quality of life in older community-dwelling women with osteoporosis. Gerontology 2002 Nov-Dec;48(6):360-8.

75. Orr R, Raymond J, Fiatarone Singh M. Efficacy of progressive resistance training on balance performance in older adults : A systematic review of randomized controlled trials. Sports Medicine 2008;38(4):317-43.

76. Bischoff-Ferrari HA, Conzelmann M, Stahelin HB, Dick W, Carpenter MG, Adkin AL, Theiler R, Pfeifer M, Allum JH. Is fall prevention by vitamin D mediated by a change in postural or dynamic balance?. Osteoporosis Int 2006;17(5):656-63.

77. Bunout D, Barrera G, Leiva L, Gattas V, de la Maza MP, Avendano M, Hirsch S. Effects of vitamin D supplementation and exercise training on physical performance in chilean vitamin D deficient elderly subjects. Exp Gerontol 2006 Aug;41(8):746-52.

78. Evatt ML, Delong MR, Khazai N, Rosen A, Triche S, Tangpricha V. Prevalence of vitamin d insufficiency in patients with parkinson disease and alzheimer disease. Arch Neurol 2008 Oct;65(10):1348-52.

79. Sato Y, Kikuyama M, Oizumi K. High prevalence of vitamin D deficiency and reduced bone mass in parkinson's disease. Neurology 1997 Nov;49(5):1273-8.

80. Sanchez B, Lopez-Martin E, Segura C, Labandeira-Garcia JL, Perez-Fernandez R. 1,25-dihydroxyvitamin D(3) increases striatal GDNF mRNA and protein expression in adult rats. Brain Research.Molecular Brain Research 2002;108(1-2):143-6.

81. Wang Y, Chiang YH, Su TP, Hayashi T, Morales M, Hoffer BJ, Lin SZ. Vitamin D(3) attenuates cortical infarction induced by middle cerebral arterial ligation in rats. Neuropharmacology 2000 Mar 3;39(5):873-80.

82. Chen KB, Lin AM, Chiu TH. Systemic vitamin D3 attenuated oxidative injuries in the locus coeruleus of rat brain. Ann N Y Acad Sci 2003 discussion 345-9; May;993:313-24.

83. Li L, Prabhakaran K, Zhang X, Zhang L, Liu H, Borowitz JL, Isom GE. 1Alpha,25-dihydroxyvitamin D3 attenuates cyanide-induced neurotoxicity by inhibiting uncoupling protein-2 up-regulation. J Neurosci Res 2008 May 1;86(6):1397-408.

84. Ibi M, Sawada H, Nakanishi M, Kume T, Katsuki H, Kaneko S, Shimohama S, Akaike A. Protective effects of 1 alpha,25-(OH)(2)D(3) against the neurotoxicity of glutamate and reactive oxygen species in mesencephalic culture. Neuropharmacology 2001 May;40(6):761-71.

85. Horak FB, Dimitrova D, Nutt JG. Direction-specific postural instability in subjects with parkinson's disease. Exp Neurol 2005 Jun;193(2):504-21.

86. Mancini M, Zampieri C, Carlson-Kuhta P, Chiari L, Horak FB. Anticipatory postural adjustments prior to step initiation are hypometric in untreated parkinson's disease: An accelerometer-based approach. European Journal of Neurology 2009 Sep;16(9):1028-34.

87. Salarian A, Horak FB, Zampieri C, Carlson-Kuhta P, Nutt JG, Aminian K. iTUG a sensitive and reliable measure of mobility. IEEE Rehabilitation Engineering in press.

88. Drouin JM, Valovich-mcLeod TC, Shultz SJ, Gansneder BM, Perrin DH. Reliability and validity of the biodex system 3 pro isokinetic dynamometer velocity, torque and position measurements. Eur J Appl Physiol 2004 Jan;91(1):22-9.

89. Watts NB, Lewiecki EM, Miller PD, Baim S. National osteoporosis foundation 2008 clinician's guide to prevention and treatment of osteoporosis and the world health organization fracture risk assessment tool (FRAX): What they mean to the bone densitometrist and bone technologist. Journal of Clinical Densitometry 2008 Oct-Dec;11(4):473-7.

90. Buatois S, Gueguen R, Gauchard GC, Benetos A, Perrin PP. Posturography and risk of recurrent falls in healthy non-institutionalized persons aged over 65. Gerontology 2006;52(6):345-52.

91. Whitney SL, Marchetti GF, Schade AI. The relationship between falls history and computerized dynamic posturography in persons with balance and vestibular disorders. Archives of Physical Medicine & Rehabilitation 2006 Mar;87(3):402-7.

92. Balance manager system - clinical interpretation guide - computerized posturography .

93. Rubenstein LZ. Falls in older people: Epidemiology, risk factors and strategies for prevention. Age & Ageing 2006 Sep;35(Suppl 2):37-41.

94. Drouin JM, Valovich-mcLeod TC, Shultz SJ, Gansneder BM, Perrin DH. Reliability and validity of the biodex system 3 pro isokinetic dynamometer velocity, torque and position measurements. Eur J Appl Physiol 2004 Jan;91(1):22-9.

95. Inkster LM, Eng JJ, MacIntyre DL, Stoessl AJ. Leg muscle strength is reduced in parkinson's disease and relates to the ability to rise from a chair. Movement Disorders 2003 Feb;18(2):157-62.

96. Allen NE, Canning CG, Sherrington C, Fung VS. Bradykinesia, muscle weakness and reduced muscle power in parkinson's disease. Movement Disorders 2009 Jul 15;24(9):1344-51.

97. Rahman S, Griffin HJ, Quinn NP, Jahanshahi M. Quality of life in parkinson's disease: The relative importance of the symptoms. Movement Disorders 2008 Jul 30;23(10):1428-34.

98. Peto V, Jenkinson C, Fitzpatrick R. PDQ-39: A review of the development, validation and application of a parkinson's disease quality of life questionnaire and its associated measures. J Neurol 1998 May;245(Suppl 1):S10-4.

99. Hagell P, Whalley D, McKenna SP, Lindvall O. Health status measurement in parkinson's disease: Validity of the PDQ-39 and nottingham health profile. Movement Disorders 2003 Jul;18(7):773-83.

100. Institute of Medicine of the National Academies. Dietary reference intakes: Calcium, phosphours, magnesium, vitamin D and fluoride. In: Washington, DC: National Academy Press; 1997. .

101. Shephard RM, Deluca HF. Plasma concentrations of vitamin D3 and its metabolites in the rat as influenced by vitamin D3 or 25-hydroxyvitamin D3 intakes. Archives of Biochemistry & Biophysics 1980 Jun;202(1):43-53.

102. Buchner DM, Hornbrook MC, Kutner NG, Tinetti ME, Ory MG, Mulrow CD, Schechtman KB, Gerety MB, Fiatarone MA, Wolf SL. Development of the common data base for the FICSIT trials. J Am Geriatr Soc 1993 Mar;41(3):297-308.

103. Ryan JW, Dinkel JL, Petrucci K. Near falls incidence. A study of older adults in the community. J Gerontol Nurs 1993 Dec;19(12):23-8.

104. Balash Y, Peretz C, Leibovich G, Herman T, Hausdorff JM, Giladi N. Falls in outpatients with parkinson's disease: Frequency, impact and identifying factors. J Neurol 2005 Nov;252(11):1310-5.

105. Fahn S, Marsden CD, Goldstein M, Calne CD, editors. Recent deveoplments in parkinson's disease. Florham Park, NJ: Macmillan Healthcare Information; 1987. .

106. May PR, Lee MA, Bacon RC. Quantitative assessment of neuroleptic-induced extrapyramidal symptoms: Clinical and nonclinical approaches. Clin Neuropharmacol 1983;6(Suppl 1):S35-51.

107. Chung KA, Lobb BM, Nutt JG, McNames J, Horak FB. Objective measurement of dyskinesia in parkinson's disease using a force plate. Mov Disord in press.

108. Washburn RA, McAuley E, Katula J, Mihalko SL, Boileau RA. The physical activity scale for the elderly (PASE): Evidence for validity. J Clin Epidemiol 1999 Jul;52(7):643-51.

109. Tombaugh TN, McIntyre NJ. The mini-mental state examination: A comprehensive review.[see comment]. J Am Geriatr Soc 1992 Sep;40(9):922-35.

110. Gill DJ, Freshman A, Blender JA, Ravina B. The montreal cognitive assessment as a screening tool for cognitive impairment in parkinson's disease. Movement Disorders 2008 May 15;23(7):1043-6.

111. Pfeifer M, Begerow B, Minne HW, Abrams C, Nachtigall D, Hansen C. Effects of a short-term vitamin D and calcium supplementation on body sway and secondary hyperparathyroidism in elderly women. Journal of Bone & Mineral Research 2000 Jun;15(6):1113-8.

112. Frisiello S, Gazaille A, O'Halloran J, Palmer ML, Waugh D. Test-retest reliability of eccentric peak torque values for shoulder medial and lateral rotation using the biodex isokinetic dynamometer. Journal of Orthopaedic & Sports Physical Therapy 1994 Jun;19(6):341-4.

113. Sato Y, Kikuyama M, Oizumi K. High prevalence of vitamin D deficiency and reduced bone mass in parkinson's disease. Neurology 1997 Nov;49(5):1273-8.

114. Hausdorff JM, Rios DA, Edelberg HK. Gait variability and fall risk in community-living older adults: A 1-year prospective study. Archives of Physical Medicine & Rehabilitation 2001 Aug;82(8):1050-6.
